# H2A.Z overexpression suppresses senescence and chemosensitivity in pancreatic ductal adenocarcinoma

**DOI:** 10.1038/s41388-021-01664-1

**Published:** 2021-02-24

**Authors:** P. A. Ávila-López, G. Guerrero, H. N. Nuñez-Martínez, C. A. Peralta-Alvarez, G. Hernández-Montes, L. G. Álvarez-Hilario, R. Herrera-Goepfert, J. Albores-Saavedra, N. Villegas-Sepúlveda, L. Cedillo-Barrón, A. E. Montes-Gómez, M. Vargas, M. Schnoor, F. Recillas-Targa, R. Hernández-Rivas

**Affiliations:** 1 Departamento de Biomedicina Molecular, Centro de Investigación y de Estudios Avanzados del Instituto Politécnico Nacional, Ciudad de México, Mexico; 2 Instituto de Fisiología Celular, Departamento de Genética Molecular, Universidad Nacional Autónoma de México, Ciudad Universitaria, Ciudad de México, Mexico; 3 Coordinación de la Investigación Científica, Red de Apoyo a la Investigación, Universidad Nacional Autónoma de México, Ciudad Universitaria, Ciudad de México, Mexico; 4 Departamento de Patología, Instituto Nacional de Cancerología, Ciudad de México, Mexico; 5 Departamento de Patología, Medica Sur Clínica y Fundación, Ciudad de México, Mexico

**Keywords:** Pancreatic cancer, Predictive markers

## Abstract

Pancreatic ductal adenocarcinoma (PDAC) is one of the most intractable and devastating malignant tumors. Epigenetic modifications such as DNA methylation and histone modification regulate tumor initiation and progression. However, the contribution of histone variants in PDAC is unknown. Here, we demonstrated that the histone variant H2A.Z is highly expressed in PDAC cell lines and PDAC patients and that its overexpression correlates with poor prognosis. Moreover, all three H2A.Z isoforms (H2A.Z.1, H2A.Z.2.1, and H2A.Z.2.2) are highly expressed in PDAC cell lines and PDAC patients. Knockdown of these H2A.Z isoforms in PDAC cell lines induces a senescent phenotype, cell cycle arrest in phase G2/M, increased expression of cyclin-dependent kinase inhibitor CDKN2A/p16, SA-β-galactosidase activity and interleukin 8 production. Transcriptome analysis of H2A.Z-depleted PDAC cells showed altered gene expression in fatty acid biosynthesis pathways and those that regulate cell cycle and DNA damage repair. Importantly, depletion of H2A.Z isoforms reduces the tumor size in a mouse xenograft model in vivo and sensitizes PDAC cells to gemcitabine. Overexpression of H2A.Z.1 and H2A.Z.2.1 more than H2A.Z.2.2 partially restores the oncogenic phenotype. Therefore, our data suggest that overexpression of H2A.Z isoforms enables cells to overcome the oncoprotective barrier associated with senescence, favoring PDAC tumor grow and chemoresistance. These results make H2A.Z a potential candidate as a diagnostic biomarker and therapeutic target for PDAC.

## Introduction

Pancreatic ductal adenocarcinoma (PDAC) represents one of the most lethal cancers in the world, primarily due to its late diagnosis and resistance to gemcitabine, the first-line chemotherapy [1, 2]. PDAC remains one of the most intractable and devastating malignancies with a median survival time of only 6 months after diagnosis and a 5-year survival rate of 5–7% [1]. Therefore, it is predicted that by 2024, PDAC will be the second cause of cancer deaths worldwide [3, 4]. Genetic alterations in PDAC have been widely studied [3, 5]. However, epigenetic mechanisms might also contribute to regulating gene expression [5, 6]. Molecular mechanisms underlying epigenetic effects have been attributed primarily to alterations in DNA methylation patterns, but also histones have emerged as critical regulators [7]. Histones can alter the epigenomic landscape mainly by two mechanisms: (1) histone post-translational modifications (PTMs) such as methylation, acetylation, ubiquitination, and phosphorylation; and (2) the substitution of canonical histones by histone variants. The incorporation of histone variants into nucleosomes is one of the most important epigenetic mechanism responsible for modifying the local structure of chromatin and regulates cellular processes such as gene expression [8, 9]. If not controlled properly, altered histone composition may contribute to cancer initiation and progression. Indeed, growing evidence links histone variants to cancer biology [7, 9]. Therefore, studying histone variant expression and functions will contribute to a better understanding of the mechanisms favoring PDAC development.

H2A.Z is a conserved histone variant found in different organisms, and it displays 60% identity with its canonical histone H2A [10]. Three histone H2A.Z variants have been identified. Two are transcribed by the non-allelic genes H2AFZ and H2AFV (called H2A.Z.1 and H2A.Z.2, respectively); and H2A.Z.2.2 is the result of alternative splicing of H2A.Z.2. However, these isoforms do not have redundant functions [11, 12]. H2A.Z is incorporated into nucleosomes and distributed to regulatory DNA elements such as promoters and enhancers and it is also present at poised promoters with bivalent chromatin [13–15]. Interestingly, H2A.Z prevents the spreading of heterochromatin [16] and is essential for the correct functioning of mouse-embryonic stem cells and the early development of different organisms [17, 18]. Depletion of H2A.Z in fibroblast cell lines induced a phenotype of senescence by allowing the overexpression of p53-dependent p21 [19]. However, the role of H2A.Z in generating a transcriptome associated with senescence in cancer is still unknown.

Given, the wide variety of functions of H2A.Z and the complexity resulting from the presence of three different isoforms, H2A.Z overexpression is crucial for the development of several cancer types [7], including breast cancer [20], bladder cancer [21], and intrahepatic cholangicarcinoma (ICC) [22]. However, most studies do not distinguish between H2A.Z isoforms. One recent study reported a unique role for the H2A.Z.2.1 isoform as a driver of malignant melanoma by promoting cell proliferation via recruitment of BRD2 and E2F1 to E2F target genes [23]. The H2A.Z.1 isoform plays a pivotal role in hepatocarcinogenesis by regulating key molecules in the cell cycle and epithelial–mesenchymal transition [24]. Here, we show that all three isoforms of histone H2A.Z are overexpressed in pancreatic tissue from PDAC patients and in PDAC cell lines. Especially overexpression of H2A.Z.1 and H2A.Z.2.1 enables cells to overcome the oncoprotective barrier associated with senescence, thus favoring the development of PDAC. Importantly, we show that depletion of H2A.Z isoforms renders PDAC cells susceptible to chemotherapy and reduces tumor growth in vivo.

## Results

### H2A.Z is overexpressed in pancreatic cancer cell lines and PDAC patient tissues

**Fig. 1:**
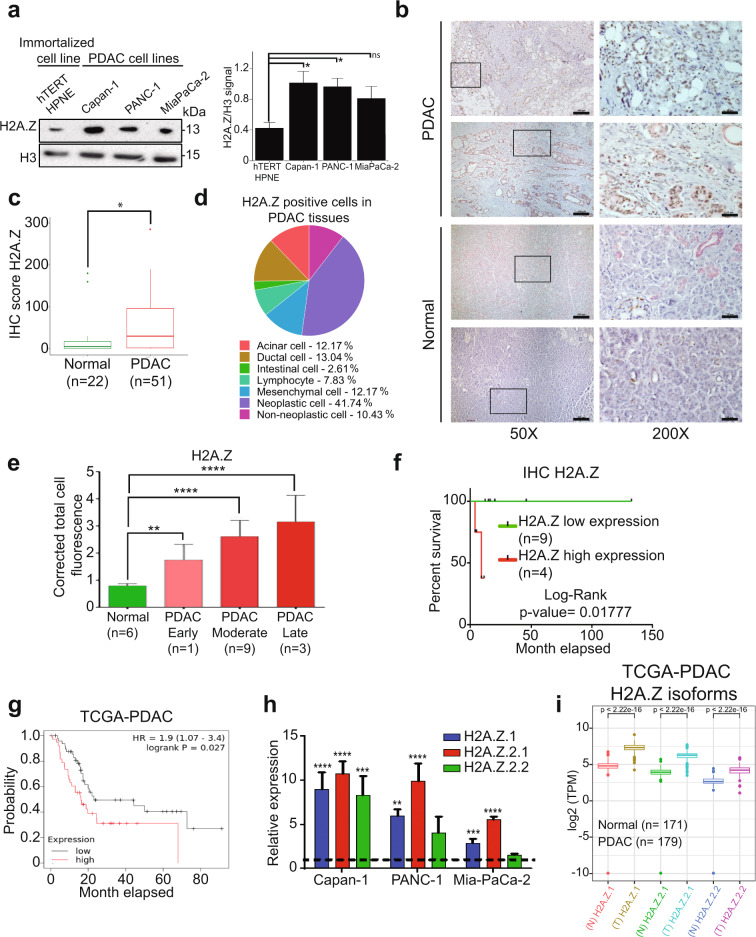
The three H2A.Z isoforms are overexpressed in PDAC cell lines and in PDAC patients. **a** Histone-enriched protein extracts from PDAC cell lines and the non-cancerous hTERT-HPNE cell line were analyzed by western blot using an anti-H2A.Z antibody. H3 was used as loading control. The graph shows the WB quantification depicting means ± SEM of four biological replicates. Statistical differences between two groups were evaluated using two-way ANOVA. *p value ≤ 0.05. **b** Normal and PDAC tissues were analyzed by IHC using an anti-H2A.Z antibody (color brown) and an anti-CK7 antibody (pink). Two representative images of PDAC and control samples at 50× are shown. The magnifications at 200× on the right correspond to the squares on the left. Scale bars = 50 μm. **c** IHC scores of H2A.Z staining in normal and PDAC tissues. Statistical differences between the two groups were evaluated by unpaired two-tailed Student’s *t* test. *p value ≤ 0.05. **d** Percentage of H2A.Z-positive cells. **e** Corrected total cell fluorescence of H2A.Z in different stages of PDAC progression. Statistical differences between two groups were evaluated using two-way ANOVA. **p value ≤ 0.01; ***p value ≤ 0.001. **f** Kaplan–Meier curves generated using the H2A.Z IHC score showing the average survival of Mexican PDAC patients. Log-Rank analysis gave a *p* value = 0.01777. **g** Kaplan–Meier curves generated using expression data from TCGA database. The graph shows the average survival of 90 PDAC patients with low levels of both H2A.Z isoforms (gray) and 53 PDAC patients with high levels of both H2A.Z isoforms (red). Log-Rank analysis gave a *p* value = 0.027. **h** Expression of the isoforms H2A.Z.1, H2A.Z.2.1, and H2A.Z.2.2 were analyzed by RT-qPCR in the cell lines CAPAN-1, PANC-1 and Mia-PaCa-2. Relative expression values normalized to the non-cancerous cell line hTERT-HPNE (dotted line). The means ± SEM of three biological replicates is shown. Two-way ANOVA analysis gave *p* values of: **p = 0.0085; ***p = < 0.001 ****p ≤ 0.0001. **i** Expression analysis of the three H2A.Z isoforms from the 179 PDAC samples available in the TCGA database, and 171 normal tissues available in the GTEx database. Statistical differences were evaluated by unpaired two-tailed Student’s *t* test.

PDAC is characterized by altered DNA methylation, histone PTMs and expression of microRNAs that all contribute to its development and progression [25]*.* However, whether histone variants contribute to PDAC development is unknown. Therefore, we first determined expression of the histone H2A.Z in PDAC using a commercial antibody, which recognizes the H2A.Z.1 and H2A.Z.2.1 isoforms, but not the H2A.Z.2.2 isoform. We found that H2A.Z was upregulated significantly in three different PDAC cell lines (Capan-1, MiaPaCa-2, and PANC-1) compared to the normal ductal pancreatic cell line hTERT-HPNE (Fig. 1a). Importantly, H2A.Z was also strongly upregulated in tissue biopsies of PDAC patients when compared to normal tissue controls. Using cytokeratin 7 (CK7) as a specific marker of ductal pancreatic cells, we could demonstrate that H2A.Z overexpression occurred specifically in these cells in 51 PDAC samples compared to 22 normal pancreas samples (Table 1) (Fig. 1b and Supplementary Fig. 1a). This result was confirmed by immunofluorescence assays performed in 13 PDAC samples and 6 control samples (Supplementary Fig. 1b). Quantification revealed a significant threefold increase of H2A.Z expression (Fig. 1c and Supplementary Fig. 1c). The majority of the H2A.Z signal in PDAC biopsies was detected in neoplastic cells (41.78%), and only 12–13% of the signal intensity was observed in each ductal, acinar and mesenchymal cells (Fig. 1d and Supplementary Table 1). Comparing H2A.Z expression levels with the degree of malignancy in 19 Mexican PDAC samples, we found a significant correlation of the highest H2A.Z levels with the highest degree of malignancy (Fig. 1e). To assess whether the overexpression of H2A.Z correlated with the survival rate, we analyzed 13 Mexican PDAC patients (those from which we had clinical information). Kaplan–Meier survival curves suggested that there is a tendency to poor survival in Mexican patients with highest H2A.Z expression levels (Fig. 1f). These results were confirmed when we analyzed data from The Cancer Genome Atlas (TCGA) (Fig. 1g). Therefore, these results suggest that overexpression of H2A.Z could participate in the regulation of gene expression involved in the development of PDAC and that H2A.Z overexpression is associated with poor survival.

**Table 1:** Clinico-pathological features of the PDAC patients.

Patient	Sex	Age (Years)	Tumor size	Metastasis	TNM stage
1	Female	20	–	–	–
2	Female	33	Tis	M0	0
3	Male	53	T3	Mx	IIB
4	Female	20	–	–	–
5	Male	78	T3	Mx	III
6	Female	69	T3	M1	IV
7	Male	76	T2	M0	IIB
8	Male	76	T2	M0	IIB
9	Male	70	T3	–	–
10	Male	73	T2	–	–
11	Female	77	T2	–	–
12	Male	40	T2	Mx	III
13	Male	83	T4		–
14	Male	75	T3	Mx	IIB
15	Female	47	T3	Mx	IIB
16	Female	70	T2	–	–
17	Female	45	T2	–	–
18	Female	42	T3	M1	IV
19	Male	61	T2	Mx	III
20	Female	56	T4	M1	IV
21	Male	69	T3	Mx	IIB
22	Female	60	-	M1	IV
23	Female	79	T3	Mx	IIB
24	Female	58	T3	Mx	IIA
25	Male	72	T3	Mx	III
26	Female	81	T3	Mx	IIB
27	Female	74	T2	–	–
28	Male	44	T3	–	–
29	Male	66	T3	Mx	IIA
30	Male	47	T3	Mx	III
31	Male	68	T2	–	–
32	Male	48	–	–	–
33	Female	58	–	–	II
34	Male	68	T2	–	–
35	Male	52	T3	Mx	IIB
36	Female	66	T1	Mx	–
37	Female	60	T3	Mx	IIA
38	Male	46	–	–	–
39	–	–	–	–	IB
40	Male	66	–	–	IIA
41	–	–	–	–	IIA
42	–	–	–	–	IIA
43	Male	74	–	–	IIA
44	Female	51	–	–	IIB
45	–	-	–	–	IIB
46	Female	57	–	–	IIB
47	–	–	–	–	IIB
48	–	–	–	–	IIB
49	–	–	–	–	III
50	–	–	–	–	III
51	–	–	–	–	IV

### All three H2A.Z isoforms are upregulated in cell lines and PDAC patient samples

To determine which of the three H2A.Z isoforms were upregulated in PDAC, we performed quantitative real-time polymerase chain reaction (RT-qPCR) using cDNA from the control cell line hTERT-HPNE and the three PDAC cell lines PANC-1, Capan-1, and MiaPaCa-2 (Fig. 1h) with specific oligonucleotides (Table 2). We found that the expression levels of the three H2A.Z isoforms were higher in all three PDAC cell lines than in hTERT-HPNE, with the isoform H2A.Z.2.1 being the most upregulated followed by the isoforms H2A.Z.1 and H2A.Z.2.2 (Fig. 1h). These results were confirmed by analyzing 179 tissue samples from PDAC samples from the TCGA database and 171 normal tissue samples from GTEx database (Genotype Tissue Expression bank) (Fig. 1i). We found for the first time that all three H2A.Z isoforms are upregulated in PDAC cell lines and, importantly, also in pancreatic tissue from PDAC patients.

**Table 2:** Oligonucleotides used for RT-qPCR.

Transcript	Sequence 5′ to 3′	Size (pb)
H2A.Z.1* Forward	GGCAGGAAATGCATCAAAAG	138
H2A.Z.1* Reverse	TGGATGTGTGGAATGACACC	
H2A.Z.2.1* Forward	GAGCTGGCAGGTAATGCTTC	147
H2A.Z.2.1* Reverse	TTTGTGGATGTGAGGGATCA	
H2A.Z.2.2 Forward	ATCAAGGCTACCATAGCTGG	133
H2A.Z.2.2 Reverse	GGTTCAGCTCAGCACACATC	
GAPDH Forward	TTTGTCAAGCTCATTTCCTGG	268
GAPDH Reverse	TGATGGTACATGACAAGGTGC	
E2F1 Forward	ACGCTATGAGACCTCACTGAA	249
E2F1 Reverse	TCCTGGGTCAACCCCTCAAG	
E2F5 Forward	CACCTTCTGGTACACAACTG	139
E2F5 Reverse	GGCTTAGATGAACTCGACTC	
CCNB1 Forward	TTGCACTTCCTTCGGAGAGC	121
CCNB1 Reverse	GAGAAGGAGGAAAGTGCACCA	
CDKN2A (P16 INKA4A) Forward	CCCAACGCACCGAATAGTTAC	135
CDKN2A (P16 INKA4A) Reverse	CACGGGTCGGGTGAGAGT	
FASN Forward	CCATGGCAACGTGATGCTAC	116
FASN Reverse	CGATGACGTGGACGGATACT	
SCD1 Forward	TCCTGGTTTCACTTGGAGCTG	95
SCD1 Reverse	GATGTGCCAGCGGTACTCAC	
IL-8 Forward	CAAGGAAAACTGGGTGCAGAG	91
IL-8 Reverse	ATTCTTGGATACCACAGAGAATGA	
IGFBP-3 Forward	CCTGGGACTCAGCACATTGA	91
IGFBP-3 Reverse	GTCCAAGCGGGAGACAGAATAT	
CTSH Forward	GGGATCCCTTACTGGATCGTG	94
CTSH Reverse	GGCCACACATGTTCTTTCCG	
DNM1 Forward	CTGCAGGTGCAGAGCGTA	134
DNM1 Reverse	GACCCAGCAGGCGGAG	
Forward H2A.Z.1 HindIII	cccaagcttATGGCTGGCGGTAAGGCT	–
Reverse H2A.Z.1 XhoI	ccgctcgagcgGACAGTCTTCTGTTGTCCTTT	

### Knockdown of the three H2A.Z isoforms causes alterations in pancreatic cell density and shape

**Fig. 2:**
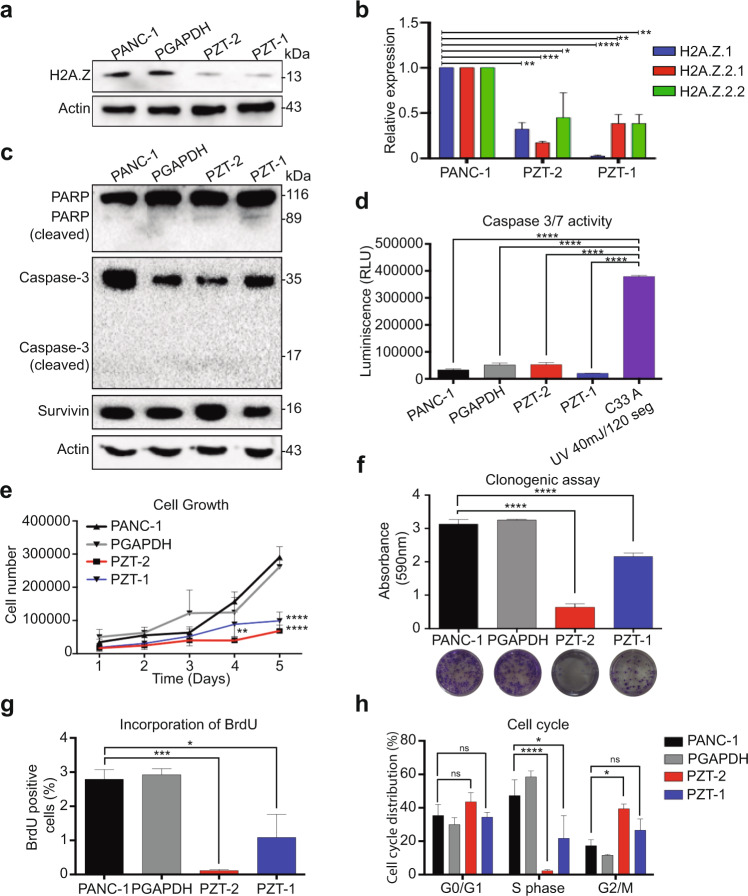
The knockdown of the three isoforms of H2A.Z induces arrest in the G2/M phase. **a** Total extracts of the non-transduced PANC-1 cell line, of the PANC-1 transduced with shRNA directed against GAPDH, and the KD clones PZT-2 and PZT-1 were analyzed by western blot using an anti-H2A.Z antibody (*n* = 3). Actin was used as loading control. **b** Expression of H2A.Z.1, H2A.Z.2.1, and H2A.Z.2.2 were analyzed by RT-qPCR in the KD clones PZT-2 and PZT-1. Values are normalized to the expression in the untransduced PANC-1 cell line. GAPDH was used as house-keeping gene. The means ± SEM of three biological replicates is shown. Significance was analyzed using two-way ANOVA: *p value ≤ 0.05; **p value ≤ 0.01; ***p value ≤ 0.001. **c** Total extracts of the PANC-1 cell line, the PGAPDH cell line and the clones PZT-2 and PZT-1 were analyzed by western blot to identify expression and cleavage of caspase 3, PARP and survivin (*n* = 3). **d** PANC-1, PGAPDH, PZT-2, and PZT-1 cell lines present low caspase 3 and 7 activity with respect to C-33A cells in which apoptosis was induced by UV light, *n* = 3. Significance was analyzed by one-way ANOVA. ****p value ≤ 0.0001. **e** Cell growth curves of PANC-1, PGAPDH, PZT-2, and PZT-1. The means ± SEM of three biological replicates is shown. Significance was analyzed by two-way ANOVA. ***p value = 0.001; ****p value ≤ 0.0001. **f** Clonogenic capacity of the cell lines PANC-1, PGAPDH, PZT-1, and PZT-2. The graph shows absorbance at 590 nm of the crystal violet retained in the colonies. The means ± SEM of three biological replicates is shown. Significance was analyzed by one-way ANOVA. ****p value ≤ 0.0001. **g** Incorporation of BrdU into the cell lines PANC-1, PGAPDH, PZT-2, and PZT-1 24 h after synchronization in G0 by serum starvation for 48 h. The means ± SEM of three biological replicates is shown. Significance was analyzed by one-way ANOVA. *p value = 0.0497. **h** Distribution of the cell line PANC-1, PGAPDH, PZT-2, and PZT-1 in cell cycle phases G0–G1, S and G2-M as examined by staining with BrdU and IP 24 h after synchronization in G0 by serum starvation for 48 h. The means ± SEM of three biological replicates is shown. Significance was analyzed by two-way ANOVA. *p value = 0.0415; ****p value ≤ 0.0001.

To determine the functional role of the overexpression of the three H2A.Z isoforms in PDAC biology, we depleted their expression levels in the cell line PANC-1 using two H2A.Z-targeting short hairpin RNAs (shRNA) [23, 26]. One shRNA decreased the expression of H2A.Z.1 by interfering with the 3′-untranslated region specific to this isoform. To deplete isoforms H2A.Z.2.1 and H2A.Z.2.2, a specific shRNA was generated targeting exon 4 (Supplementary Fig. 2a and Table 3). Depletion was confirmed by RT-qPCR and western blot (WB) (Supplementary Fig. 2b, c). Four knockdown (KD) clones were obtained with different depletion levels of H2A.Z isoforms (Supplementary Fig. 2d, e). Of the four KD H2A.Z clones obtained we chose the KD clones PZT-2 and PZT-1 because they showed the lowest mRNA and protein levels for the three H2A.Z isoforms (Fig. 2a, b and Supplementary Fig. 2d, e) and the lowest proliferative capacity (Supplementary Fig. 2f, g). Both KD clones showed a mostly round cell morphology, while other cells were rather flat with extensions (Supplementary Fig. 2h). Interestingly, cell numbers were reduced significantly after 72 h of culture compared to that in control PANC-1 cells (Supplementary Fig. 2h). A shRNA against GAPDH (PGAPDH) was used as specificity control (Supplementary Fig. 2i). Therefore, all these data suggest that the three H2A.Z isoforms are involved in regulating the PDAC development.

**Table 3:** Oligonucleotides used to generate sh against H2A.Z isoforms, GAPDH and scramble.

Name	Sequence of sense strand 5′ to 3′
shRNA_H2A.Z.1^a^	TGCTTCAAAGAAGCTATTGATT
shRNA_H2A.Z.2.1y2.2^a^	TGTCTCTTATCAAGGCTACCATA
shRNA_GAPDH #1	GCCAGGTGGTCTCCTCTGACTTA
shRNA_SCR1	GGATAATTCGTTAAGTACATCT
shRNA_SCR2	GCACTCCTATTATGCAATGTACT
Loop sequence	TTCAAGAGA
Terminal sequence	TTTTTTGGAAAC

^a^[26].

### Depletion of H2A.Z isoforms promotes G2/M phase cell cycle arrest in pancreatic cells

Diminished proliferation of the KD clones could be explained by increased apoptosis or cellular senescence. However, we did not observe any significant difference neither in the processing of PARP nor caspase 3 (Fig. 2c and Supplementary Fig. 2j). Moreover, luminescence assays did not show any significant overactivation of caspases 3 and 7 in the H2A.Z-depleted cells (Fig. 2d); and all cells did express survivin (Fig. 2c), an inhibitor of apoptosis. Thus, apoptosis in H2A.Z-depleted cells is not the mechanism responsible for decreased cell numbers.

As expected, growth curves for PANC-1, PGAPDH, PZT-1, and PZT-2 cells over 5 days showed lower rates of cell proliferation over time for both KD clones compared to control cells (Fig. 2e), which was confirmed by clonogenic assays (Fig. 2f), suggesting that decreased expression of the H2A.Z isoforms affects DNA replication. Indeed, BrdU incorporation rates were significantly lower in KD cells than in control cells (Fig. 2g). To analyze the cell cycle, we performed BrdU and propidium iodide staining assays (Fig. 2h and Supplementary Fig. 2k). We found that clone PZT-2 and PZT-1 showed very little replication (1.42% and 20% cells in S phase, respectively) compared to PANC-1 and PGAPDH cells (50% and 60%, respectively). Interestingly, most of the H2A.Z-depleted cells were arrested in the G2/M phase. Thus, these results clearly show that depletion of the three H2A.Z isoforms promote cell cycle arrest, but not apoptosis, suggesting the possibility that senescence could be the mechanism responsible for reduced DNA replication.

### H2A.Z isoform depletion promotes senescence

**Fig. 3:**
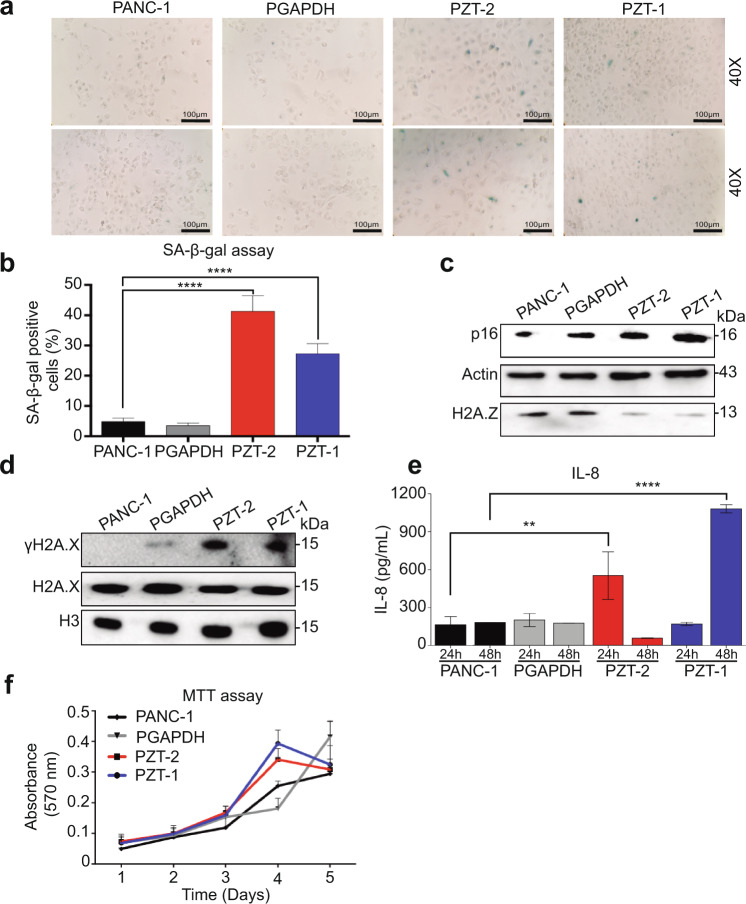
The knockdown of the three H2A.Z isoforms induce cellular senescence. **a** SA-β-galactosidase activity in PANC-1, PGAPDH, PZT-2, and PZT-1 show senescent cells in blue. Two representative images at 20× and 40× are shown. Scale bar = 100 μm. The means ± SEM of three biological replicates is shown. **b** Quantification of senescent cells positive for SA-β-galactosidase. Significance was analyzed by one-way ANOVA. ****p value ≤ 0.0001. **c**, **d**. Total extracts of PANC-1, PGAPDH, PZT-2, and PZT-1 cell lines were analyzed by western blot using antibodies against the cell cycle inhibitor p16, and H2A.X and γH2A.X, which are associated with DNA damage. Actin was used as loading control (*n* = 3). **e** IL-8 levels in cell culture supernatants of PANC-1, PGAPDH, PZT-2, and PZT-1 cells after 24 and 48 h. The means ± SEM of three biological replicates are shown. Significance was analyzed by one-way ANOVA. **p value = 0.0045. **f** Kinetics of metabolic activity in PANC-1, PGAPDH, PZT-2, and PZT-1 cells as determined by MTT assay. The means ± SEM of three biological replicates is shown. Two-way ANOVA did not yield any significant difference (*n* = 3).

To determine whether reduced DNA replication is associated with senescence, we measured SA-β-galactosidase activity and found that both KD clones had higher SA-β-galactosidase activity than the control cell lines (Fig. 3a, b). In addition, increased expression of p16 and higher levels of phosphorylation of the histone variant H2A.X (γH2A.X) were observed in both clones (Fig. 3c, d). Of note, levels of secreted interleukin 8 (IL-8), a pro-inflammatory chemokine and marker of the senescence-associated secretory phenotype (SASP), were higher in the KD clone PZT-2 after 24 h and in the KD clone PZT-1 after 48 h (Fig. 3e). Senescent cells are also characterized by remaining metabolically active [27]. Thus, we analyzed metabolic activity using MTT assays and found that both KD clones showed metabolic activities over time like the control cell lines (Fig. 3f). These data strongly suggest that depletion of H2A.Z isoforms causes senescence.

### Knockdown of H2A.Z isoforms alters expression of genes involved in senescence

**Fig. 4:**
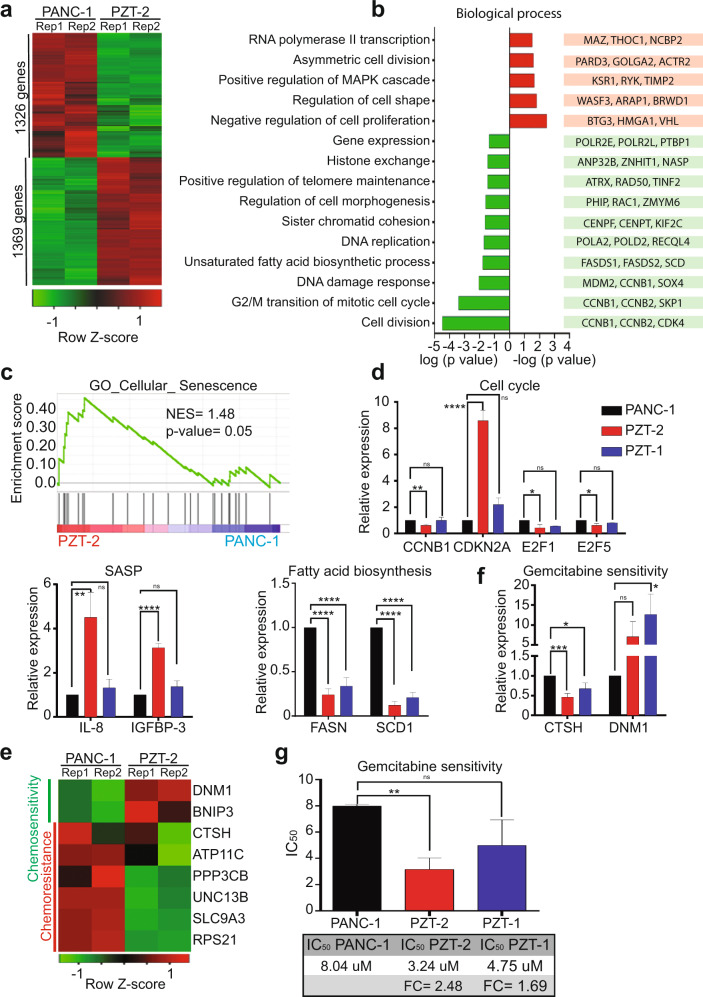
H2A.Z regulates the expression of genes associated with senescence and resistance to gemcitabine. **a** RNA-seq heatmap of the expression profile of the 2695 differentially expressed genes in PANC-1 and PZT-2. The differential gene expression presents a false discovery rate (FDR) < 0.05. Overexpressed genes are shown in red and downregulated genes are shown in green. Two biological replicates are shown. **b** Ontology of genes (biological processes) of the differential gene expression in PZT-2. The overexpressed biological processes are shown in red and the diminished biological processes are shown in green. The enrichment groups have a *p* value < 0.05. Representative genes of each process are shown. **c** Gene set enrichment analysis (GSEA) of altered genes in PZT-2. A gene set associated with cellular senescence (GO_CELLULAR_SENESCENCE, Systematic name: M11558) is enriched in PZT-2. FDR = 0.05; FWER *p* value = 0.02. **d** Validation of RNA-seq by RT-qPCR for genes associated with senescence. The values are normalized to the untransduced PANC-1 cell line. GAPDH was used as house-keeping gene. The means ± SEM of three biological replicates is shown. Significance was analyzed by two-tailed Student’s *t* test. *p ≤ 0.05; **p = 0.0011; ***p = 0.0006; ****p ≤ 0.0001. **e** Heatmap of the differential gene expression profile in PANC-1 and PZT-2 associated with chemoresistance. The differential gene expression presents an FDR < 0.05. Overexpressed genes are shown in red and reduced genes in green. Two biological replicates are shown. **f** Validation by RT-qPCR for the genes DNM1 and CTSH. The values are normalized to the untransduced PANC-1 cell line. GAPDH was used as house-keeping gene. The means ± SEM of three biological replicates is shown. Significance was analyzed by two-tailed Student’s *t* value ***p = 0.0004. **g** Cytotoxicity assay using different gemcitabine concentrations in PANC-1, PZT-1, and PZT-2 cells after 72 h. IC50 values of three biological replicates with gemcitabine are shown. Significance was analyzed by two-tailed Student’s *t* value **p ≤ 0.01.

To determine which genes are dysregulated by the depletion of the H2A.Z isoforms, we performed RNA sequencing (RNA-seq) of PANC-1, scrambled shRNA (PSCR) cells, (as specificity control), and the KD clone PZT-2 that showed the strongest senescent phenotype. As expected, we found that both controls (PSCR and PGAPDH) had a similar phenotype that the parental PANC-1 cell line (Supplementary Fig. 3a, 3b) and that differently expressed genes were similar in PANC-1 vs PZT-2 and PZT-2 vs PSCR (Fig. 4a and Supplementary Fig. 3). The heatmap clearly showed differences in the transcriptional profiles between PANC-1 and PZT-2 cells, with 1369 upregulated genes and 1326 downregulated genes in PZT-2 cells (Fig. 4a). The gene ontology (GO) analysis indicated that the downregulated transcripts participate primarily in cell division, nuclear division, G2/M transition, and DNA replication (Fig. 4b), thus further supporting the idea that decreased expression of H2A.Z isoforms promotes senescence. These processes were also changed when comparing PSCR vs PTZ-2 (Supplementary Fig. 3c). The upregulated transcripts are also involved in senescence-associated biological processes such as negative regulation of proliferation and asymmetric cell division (Fig. 4b). Gene set enrichment analysis using a senescence signature to identify the senescence-related gene set showed a strong enrichment of senescence genes in PZT-2 cells compared to PANC-1 cells (Fig. 4c). In summary, all data clearly demonstrate that decreased levels of H2A.Z isoforms promote senescence.

To validate the RNA-seq data, we performed RT-qPCR assays for some of the identified mRNAs. We show that several genes involved in the cell cycle such as cyclin B1, CDKN2A, E2F1, and E2F5 were significantly downregulated (Fig. 4d), while p16 increased in both KD clones (Fig. 4d), thus corroborating the RNA-seq and WB data (Fig. 3c). Moreover, our RNA-seq data showed that the fatty acid pathway was also significantly downregulated in both KD clones. Senescent cells show a profound modification of fatty acid biosynthesis and fatty acid desaturation [28]. Fatty acid synthase (FASN) and stearoyl-CoA-desaturase 1 (SCD1), enzymes converting the saturated fatty acids palmitic and stearic acids to their monosaturated forms palmitoleic and oleic acids, are significantly decreased in senescent cells [28]. Thus, we analyzed FASN and SCD1 and detected decreased expression in both KD clones (Fig. 4d). Similar results were observed when comparing the expression level of CCNB1, E2F5, IL-8, SCD1, CTSH, and DNM1 genes between PSCR vs PTZ-2 (Supplementary Fig. 3d). In conclusion, our RNA-seq and RT-qPCR data confirm that decreased H2A.Z levels promote a senescent phenotype. A summary of the pathways affected in the absence of H2A.Z is shown in Supplementary Fig. 4, Supplementary Tables 2 and 3.

### Reduced expression of H2A.Z isoforms reverses chemoresistance

To date, gemcitabine is the most commonly used chemotherapeutic agent in PDAC, and genes that confer resistance to this drug have already been identified [29]. Analyzing our RNA-seq data, we identified altered expression of eight of these mRNAs in the clone PZT-2. Dynamin-1 (DNM1) and Bcl-2-interacting protein 3, that are known to be upregulated in gemcitabine-sensitive cells [29], were also upregulated in the clone PZT-2 (Fig. 4e). In addition, low expression levels of CTSH, ATP11C, PPP3CB, UNC13B, SLC9A3, and RPS21 were detected in the clone PZT-2. These six genes have been reported to be upregulated in gemcitabine-resistant cells [29]. As shown in the heatmap of Fig. 4e, the expression trends of these mRNAs in the clone PZT-2 were opposite to those in PANC-1 cells that are resistant to gemcitabine. To test whether this holds true in our cells, RT-qPCR assays were performed using primers for DNM1 and CTSH. We found that both KD clones indeed showed significantly reduced expression of CTSH, whereas DNM1 was significantly increased (Fig. 4f and Supplementary Fig. 3d). This finding suggests that reduced expression of H2A.Z reverses chemoresistance to gemcitabine. To test this idea, we grew both cell lines in the presence of gemcitabine and found that clones PZT-2 and PZT-1 were significantly more sensible to gemcitabine (2.48 vs 1.69 times, respectively) than PANC-1 (Fig. 4g). In conclusion, H2A.Z regulates the expression of genes involved in chemoresistance, thus controlling the sensitivity of pancreatic tumor cells to gemcitabine therapy.

### Depletion of H2A.Z isoforms decreases tumor growth in vivo

**Fig. 5:**
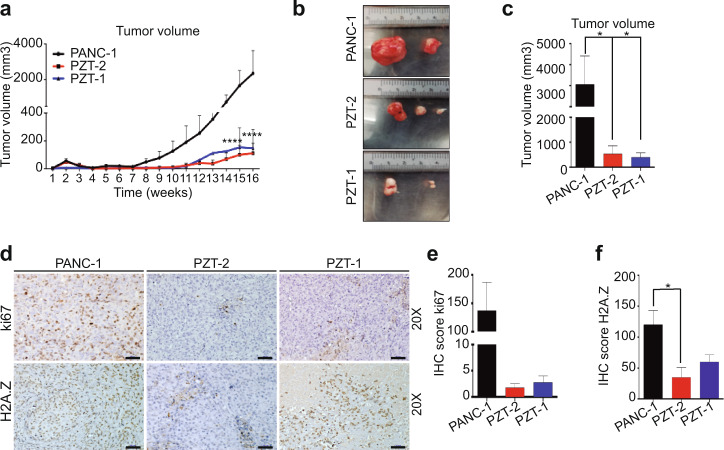
Depletion of H2A.Z decreases tumor growth. **a** Weekly tumor volume from three mice per group subcutaneously injected with 5 × 10^6^ PANC-1, PZT-1, and PZT-2 cells. The means ± SEM of three inoculated mice is shown. Significance was analyzed by two-way ANOVA. *p value = 0.0017; **p value = 0.0189. **b** Representative images of the tumors from mice injected with PANC-1, PZT-1, and PZT-2 cells. *n* = 2. **c** Graph showing the final tumor volume obtained from mice injected with PANC-1, PZT-1, and PZT-2. Means ± SEM are shown. Significance was analyzed by two-tailed Student’s *t* test. *n* = 2. **d** Tumors derived from PANC-1, PZT-1, and PZT-2 cells were immunostained for Ki67 and H2A.Z. H2A.Z knockdown tumors showed lower Ki67 and H2AZ staining than tumors derived from PANC-1 cells. Scale bars = 50 μm. **e**, **f**. Immunohistochemical score of Ki67 and H2A.Z signals in the tumors. Statistical differences between the two groups were evaluated by unpaired two-tailed Student’s *t* test. *n* = 2.

To examine the effect of H2A.Z depletion on tumor growth in vivo, the KD clones and PANC-1 cells were subcutaneously injected into nu/nu mice. We found that tumors derived from both KD clones had significantly lower tumor volume than those derived from PANC-1 cells (Fig. 5a–c). Tumors derived from both KD clones showed less Ki67 and H2A.Z staining than tumors derived from PANC-1 cells (Fig. 5d–f) indicating that depletion of H2A.Z isoforms decelerates tumor growth.

### H2A.Z.1 and H2AZ.2.1 overexpression partially restores the oncogenic phenotype

**Fig. 6:**
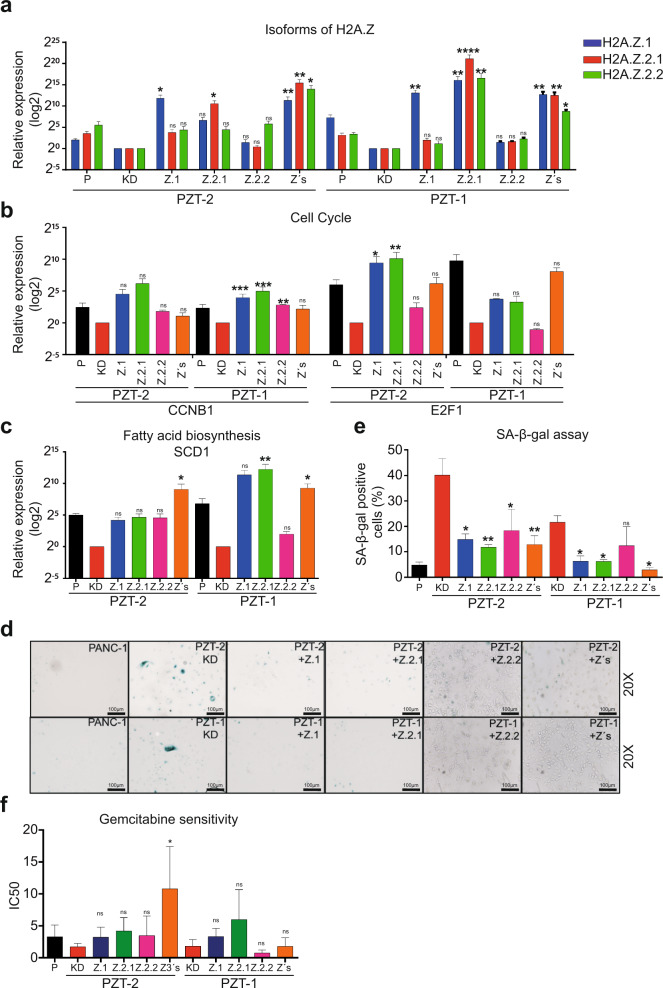
Overexpression of H2A.Z isoforms affects the oncogenic phenotype of PDAC cells. **a** Expression of the three H2A.Z isoforms analyzed by RT-qPCR in the KD cell lines PZT-1 and PZT-2 transfected with plasmids coding for H2A.Z.1, H2A.Z.2.1, and H2A.Z.2.2 alone or together (Z3s). The values are normalized to the non-transfected PZT-2 and PZT-1 KD cell lines. GAPDH was used as house-keeping gene. The means ± SEM of three independent experiments is shown. Expression of CCNB1, E2F1 (**b**) and SCD1 (**c**) analyzed by RT-qPCR in the KD clones PZT-1 and PZT-2 transfected with H2A.Z.1, H2A.Z.2.1, and H2A.Z.2.2 alone or together as analyzed in (**a**). *n* = 3. **d** SA-β-galactosidase assay performed in the knockdown clones PZT-1 and PZT-2 overexpressing either H2A.Z.1, H2A.Z.2.1, and H2A.Z.2.2 alone or all isoforms together (Z3s). Representative images at 20× are shown. Scale bars = 100 μm. **e** Quantification of the SA-β-galactosidase assays (means ± SEM of three independent experiments). **f** Cytotoxicity assay using gemcitabine in the KD clones PZT-1 and PZT-2 transfected with H2A.Z.1 H2A.Z.2.1, and H2A.Z.2.2 alone or together. Cells were incubated in logarithmic concentrations of gemcitabine for 72 h. The IC_50_ of three biological replicates in two independent experiments are shown. ANOVA analysis gave *p* values of: *p ≤ 0.05; **p ≤ 0.01; ***p ≤ 0.001 ****p ≤ 0.0001.

To confirm that overexpression of the three H2A.Z isoforms are responsible for avoiding senescence, both KD cells were co-transfected with the plasmids pCDNA3-H2A.Z.1 that encodes for the H2A.Z.1 isoform, pH2A.Z.2.1-Myc that encodes for H2A.Z.2.1, and pH2A.Z.2.2-Myc that encodes for H2A.Z.2.2. In addition, KD clones were only transfected with one isoform to identify which of the three H2A.Z isoforms plays a more important role in re-establishing the oncogenic phenotype. RT-qPCR and WB assays confirmed that after transfection with the respective plasmids, mRNAs and proteins were increased (Fig. 6a and Supplementary Fig. 5a–c).

Interestingly, we found that overexpression of three H2A.Z isoforms alone or together did in most cases increase the expression of cyclin B1 and E2F1 (Fig. 6b). Of note, only overexpression of all three isoforms was able to significantly increase SCD1 expression in both KD clones, whereas overexpression of single isoforms only caused non-significant trends toward higher SCD1 expression (except for H2A.Z.2.1 alone in clone PZT-1) (Fig. 6c). As expected, SA-β-galactosidase activity was significantly lower in both KD clones after overexpression of the three H2A.Z isoforms alone or together (Fig. 6d, e); with the only exception being H2A.Z.2.2 that only showed a non-significant tendency toward lower activity. Finally, the chemoresistance phenotype was significantly reversed when all three isoforms were overexpressed together in the clone PZT-2 (Fig. 6f). These data suggest that overexpression of H2A.Z isoforms contributes to an oncogenic phenotype in PDAC.

## Discussion

Epigenetic dysregulation has emerged as a key player in the regulation of carcinogenic processes [30, 31]. Increasing experimental evidence suggests that the incorporation of histone variants into the nucleosome can generate completely different regulatory states, which creates a unique chromatin state that controls specific functions dependent on the histone variant [32, 33]. In addition, it has also been shown that the exchange of canonic histones with histone variants can contribute to the onset and progression of cancer [7]. This exchange has already been demonstrated for the histone variant H2A.Z in different types of cancer such as breast, bladder, and ICC [20–22]. In this study, we found that overexpression of the H2AZ.1 and H2A.Z.2.1 isoforms favor the development of PDAC by avoiding senescence. Although the transcriptional levels of H2A.Z.2.2 were also increased in PDAC cell lines and PDAC patients, our rescue data suggest that this isoform has a less important role in PDAC development. This finding might be explained by the fact that H2A.Z.2.2-containing nucleosomes are less stable than nucleosomes that contain H2A.Z.1 and H2A.Z.2.1 [12]. Interestingly, our rescue data also revealed that overexpression of one H2A.Z isoform increased the mRNA expression levels of the other isoforms suggesting a feed-back regulatory loop. Thus, H2A.Z.1 and H2A.Z.2 could be mutually incorporated in the promoter region of both genes and replace each other, which may also explain the compensation between the two isoforms. This idea is supported by the recent finding that many genes are regulated by binding of both H2A.Z.1 and H2A.Z.2.1 to the same promoters in untransformed human fibroblast and cell tumoral U2OS [34].

Although a relation of H2A.Z depletion with senescence has previously been shown in human fibroblast cells [19, 35, 36], we show for the first time that this mechanism blocks cancer progression, specifically in PDAC. Our findings regarding H2A.Z isoform expression differ from other reports. For example, in malignant melanoma, H2A.Z.1 is the predominant isoform followed by H2A.Z.2.1 [23], whereas in liver cancer only the H2A.Z.1 isoform is overexpressed [24]. We show now that mainly H2A.Z.1 and H2A.Z.2.1 isoforms are necessary for PDAC development. Therefore, it is crucial to unravel the role of each H2A.Z isoform in different types of cancers and it is tempting to speculate that different organ-specific transcription factors may be responsible for controlling the expression of each H2A.Z isoform in a given context. In agreement with this idea, MCF-7 breast cancer cells treated with estrogen E2 favor the enrichment of ERα and MYC at the H2AFZ gene promoter to trigger H2A.Z expression [20].

Given the relation of H2A.Z and senescence in fibroblasts [19, 35, 36], we studied senescence in H2A.Z-depleted PDAC cells. Our rescue data suggest that the loss of H2A.Z.1 and H2A.Z.2.1 and to a lesser extent also H2A.Z.2.2 favors a senescent phenotype. Cellular senescence has long been thought to be induced by an irreversible cell cycle arrest in G1 phase due to the accumulation of the cyclin-dependent kinase inhibitors p21 and p16, which block the phosphorylation of the retinoblastoma tumor suppressor to stop DNA replication [37]. More recently, it has been shown that repression of p300 HAT activity induces histone H3 and H4 hypoacetylation leading to a senescence-like growth arrest in G2/M; and that downregulation of p300 HAT activity leads to p53, p21, and p16-independent senescence [38]. In line with this finding, we found that depletion of the two H2A.Z genes reduced proliferation due to a G2/M phase arrest. A possible explanation could be the low transcription level of one of the key regulators of entry into mitosis, cyclin B1, because transcriptional suppression of cyclin B1 is known to generate a G2/M phase arrest [39]. Moreover, cyclin B1 is degraded in response to ATM/ATR‐dependent DNA damage during this arrest [40]. This agrees with our results that depletion of H2A.Z promotes DNA damage, as evidenced by increased levels of γH2A.X. Thus, we propose that depletion of H2A.Z in PDAC cells favors the accumulation of DNA damage, and consequently a reduction in the transcription of cyclin B1 causing G2/M cell cycle arrest and senescence. By contrast, other hallmarks of senescence such as SAHF (senescence-associated heterochromatin focus assembly) were not observed in our KD clones after staining with DAPI and H3K9me3, probably because SAHF is not a common feature of cellular senescence [41]; although SAHF formation occurs in cultured cells under oncogenic stress, genotoxic stress, and replicative senescence [41]. SASP is another feature associated with senescence, with overexpression of IL-8 and IL-6 being common markers [41]. While we observed overexpression of IL-8 and IGFBP-3, two proteins implicated in SASP, IL-6 overexpression was not detected. A possible explanation could be that the senescent secretome is a heterogeneous mix of proteins depending not only on the stage of senescence progression, but also on the affected cell type and the nature of the inducing cue. Therefore, all these data indicate that depletion of H2A.Z isoforms allows PDAC cells to acquire a senescence phenotype leading to tumor suppression. Thus, we discovered a new function of H2A.Z not described previously in other types of cancer [41].

PDAC is highly resistant to gemcitabine [2]. We found that depletion of two H2A.Z genes decrease the expression of genes involved in resistance and sensitivity including genes involved in nucleoside metabolism, cell cycle regulation, and apoptosis [29], overall leading to increased sensitivity to chemotherapy. Furthermore, our rescue experiments suggest that H2A.Z isoforms could play a role in regulating gemcitabine chemoresistance. This result is consistent with previous data showing that H2A.Z sensitizes melanoma and ICC cells to chemotherapy [22, 23]. However, in those studies the genes implicated in the regulation of chemotherapy sensitivity were not identified [22, 26]. In particular, in ICC, depletion of the H2A.Z.1 isoform increases efficiency of cisplatin to induce apoptosis by increasing expression of caspases 3 and 9 [22]. By contrast, pancreatic cell lines resistant to gemcitabine showed hyper-activated PI3K/AKT signaling, but not increased apoptosis suggesting that the mechanisms inducing chemotherapy resistance is different depending on the type of cancer and drug [29]. This is important considering that H2A.Z incorporation into nucleosomes is a reversible modification. Consequently, the development of an epigenetic drug that targets H2A.Z may reverse this epigenetic modification and decrease the detrimental effect produced by its overexpression.

We found that H2A.Z is strongly upregulated in pancreatic cancer cell lines, but importantly, also in neoplastic cells of PDAC patients, where it significantly correlated with the degree of tumor malignancy. High H2A.Z levels are associated with a greater proliferative capacity of tumor cells in different types of cancer [20]; and in PDAC as well. These findings suggest that H2A.Z directly drives tumor aggressiveness. In line with this, in breast cancer, high levels of H2A.Z correlate with cell cycle regulators suggesting a direct connection between high levels of H2A.Z and high proliferative capacity of tumor cells [20]. Therefore, a therapeutic strategy to decrease H2A.Z expression may stop or at least delay the progression of those cancers.

Kaplan–Meier survival curves revealed a significant correlation of high H2A.Z levels with shorter survival in PDAC patients, suggesting that this epigenetic alteration may serve as a biomarker of poor prognosis for PDAC. Given that H2A.Z together with other epigenetic markers such as 5MC, H2AK119Ub, and H3K4me2 have been detected in cell-free nucleosomes in serum samples from PDAC patients [42], H2A.Z levels could even be a useful diagnostic tool using non-invasive procedures.

In conclusion, our results suggest that overexpression of the H2A.Z isoforms generates a transcriptional profile that allows cells to overcome the oncoprotective barrier associated with senescence, favoring the development of PDAC, tumorigenesis and the generation of chemoresistance. Thus, the histone variant H2A.Z could be used as a promising diagnostic biomarker and pharmacological target in PDAC therapy.

## Methods

### Tissue samples

Formalin-fixed and paraffin-embedded tissues from 51 PDAC surgical specimens and 22 non-cancerous pancreatic tissue samples adjacent to the tumor from post-mortem studies were provided by the Department of Pathology at Instituto Nacional de Cancerología (INCan, Mexico City, Mexico). Tissues were collected over a period of 12 years (2006–2017). Clinical features are shown in Table 1. Samples were used for immunohistochemistry assays as described below.

This study was approved by the Center for Research and Advanced Studies (CINVESTAV) Committee of Bioethics for Human Research (035/2016) and by the Research and Ethic on Research Committee of INCan (approval number: INCAN/CI/1110/17).

### Cell culture and antibodies

The PDAC cell lines PANC-1 (ATCC^®^ CRL-1469^TM^), Capan-1 (ATCC^®^ HTB-79^TM^), MiaPaCa-2 (ATCC^®^ CRL-1420^TM^), and the non-cancerous pancreatic ductal cell line hTERT-HPNE (ATCC^®^ CRL-4023^TM^) were purchased from the American Type Culture Collection (ATCC) and cultured according to their protocols. The hTERT-HPNE cell line was grown in DMEM medium without glucose (Sigma D-5030), supplemented with 25% M3 Base medium (Incell Corp M300F-500), 2 mM l-glutamine, 1.5 g/L sodium bicarbonate, 5% fetal bovine serum (FBS), 10 ng/mL human recombinant EGF, 5.5 mM d-glucose and 750 ng/mL puromycin. The MiaPaCa-2 and PANC-1 cell lines were grown in DMEM medium (ATCC^®^ 30-2002^TM^) supplemented with 10% FBS. Capan-1 cells were grown in IMDM medium (ATCC^®^ 30-2005^TM^), supplemented with 20% FBS. The cell lines were cultured in a humid atmosphere, with 5% CO_2_ at 37 °C. The absence of mycoplasm was confirmed by PCR.

All the antibodies used in this work are indicated in Table 4.

**Table 4:** Primary and secondary antibodies used for this study.

Antibody	Molecular weight	Source	Dilution	Manufacturer
H2A.Z	13 kDa	Rabbit	WB: 1:1,000	Cell Signaling 2718
			IHQ: 1:100	
Cytokeratin 7	54 kDa	Mouse	IHQ: 1:100	Dako M7018
PARP	116/89 kDa	Rabbit	WB: 1:1000	Cell Signaling 9542
Caspase 3	17/19/35 kDa	Rabbit	WB: 1:1000	Cell Signaling 9662
Survivin	16 kDa	Rabbit	WB: 1:1000	Cell Signaling 2808
p16	16 kDa	Mouse	WB: 1:200	Santa Cruz
AKT	60 kDa	Rabbit	WB: 1:1000	Cell Signaling 9272
AKT-p	60 kDa	Rabbit	WB: 1:1000	Cell Signaling 4060
ERK	42/44 kDa	Rabbit	WB: 1:1000	Cell Signaling 9102
ERK-p	42/44 kDa	Rabbit	WB: 1:1000	Cell Signaling 9101
H2A.X	15 kDa	Rabbit	WB: 1:1,000	Abcam ab11175
γH2A.X	15 kDa	Rabbit	WB: 1:1,000	Cell Signaling 2577
GAPDH	36 kDa	Rabbit	WB: 1:20,000	GeneTex GTX100118
Actina	43 kDa	Mouse	WB: 1:5000	Millipore MAB1501
H3-COOH	15 kDa	Rabbit	WB: 1:150,000	Abcam ab1791
Myc-tag		Mouse	WB: 1:500	Cell Signaling 2276
BrdU	–	Mouse	CF: 1:100	BD Pharmingen 555627
Secondary antibodies				
HRP anti-Rabbit		Goat	WB: 1:7,500	Zymed 81-6120
HRP anti-Mouse		Goat	WB: 1:7,500	Zymed 81-6520
anti-Mouse FITC		Goat	CF: 1:200	Zymed 81-6511

### Statistical analysis

Data are presented as the mean ± standard error (SEM). The GraphPad Prism v6.01 software (GraphPad Software, San Diego, CA) was used to perform all statistical analyzes. To compare more than two groups, a one-way ANOVA was performed. To compare two groups, Student’s *t* test was performed. A *p* value < 0.05 was considered statistically significant.

## Supplementary information

Supplementary Figure 1.

Supplementary Figure 2.

Supplementary Figure 3.

Supplementary Figure 4.

Supplementary Figure 5.

Supplementary Table 1.

Supplementary Table 2.

Supplementary Table 3.

Supplementary Figures

Supplementary Tables

Supplementary Methods

## Data Availability

The accession number for RNA-seq data sets reported in this paper is NCBI GEO: GSE129182. The authors declare that all other data supporting the findings of this study are available within the article and its supplementary information files.
