# Supplementary figures and images for: H2A.Z overexpression suppresses senescence and chemosensitivity in pancreatic ductal adenocarcinoma
Source: Oncogene. 2021 Feb 24;40(11):2065–80. doi: 10.1038/s41388-021-01664-1 (PMC7979544; doi:10.1038/s41388-021-01664-1)

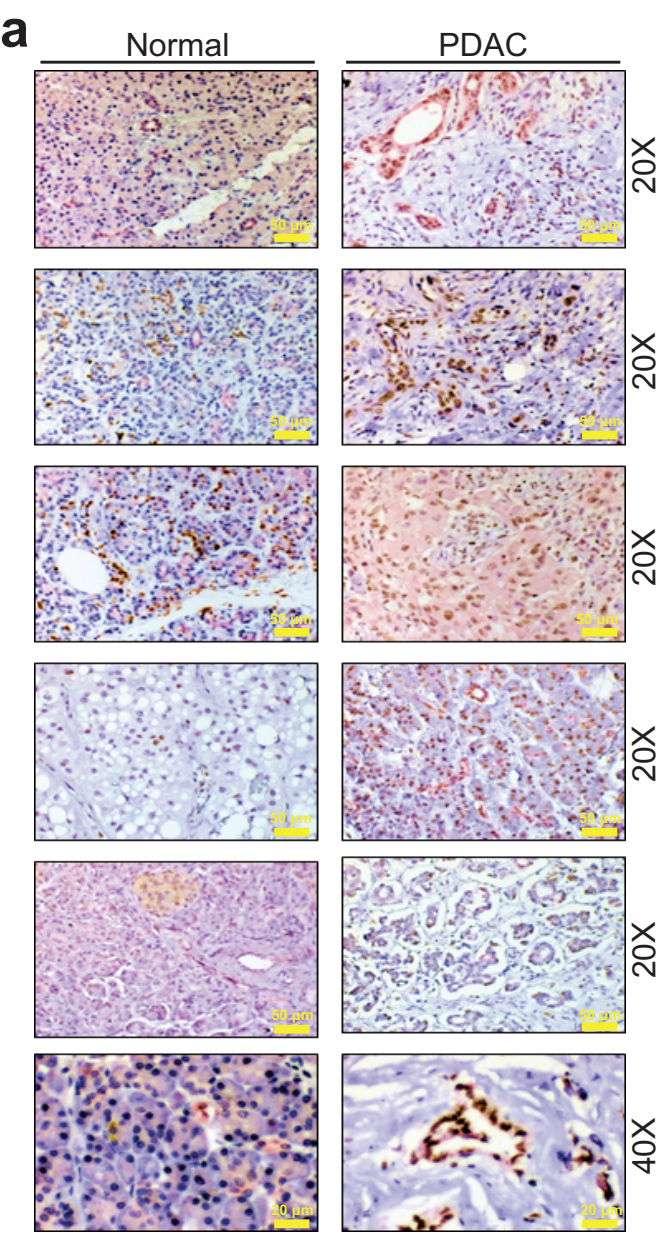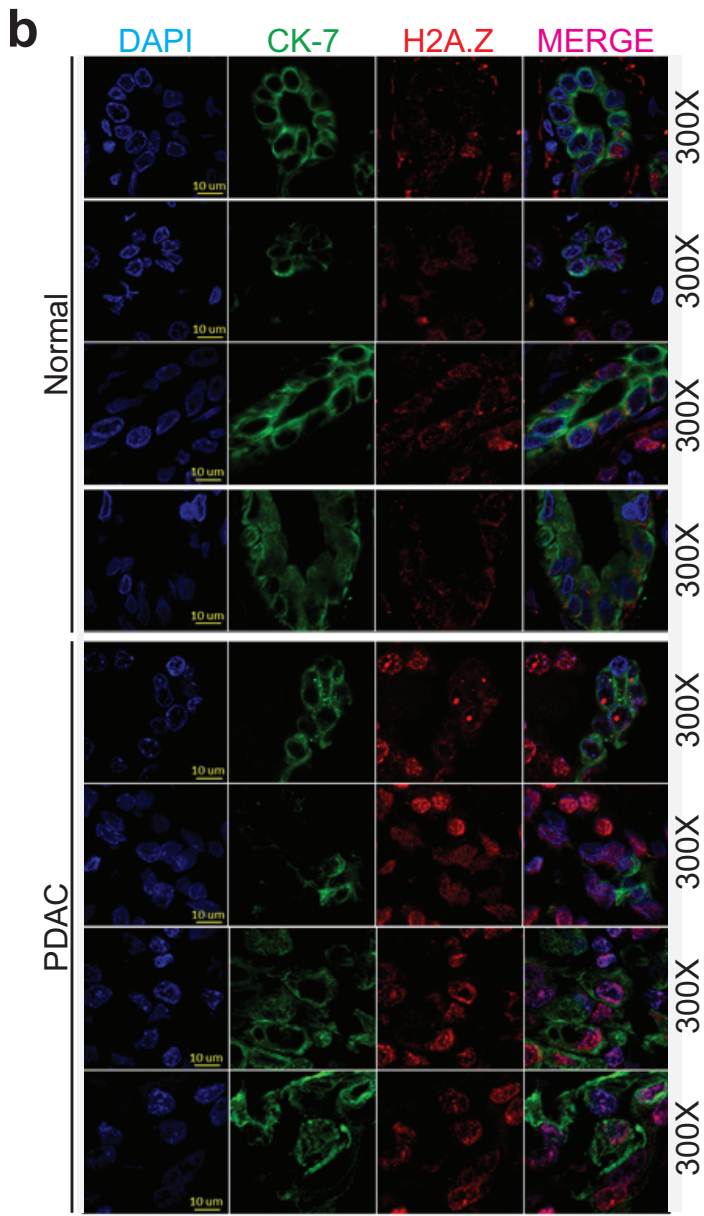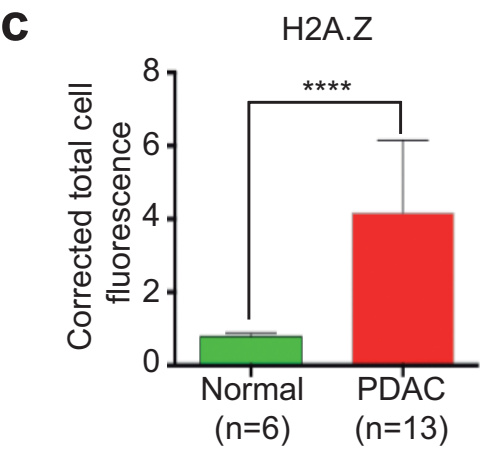

Supplement: Supplementary file 1 — Supplementary Figure 1. [file 41388_2021_1664_MOESM1_ESM.pdf]

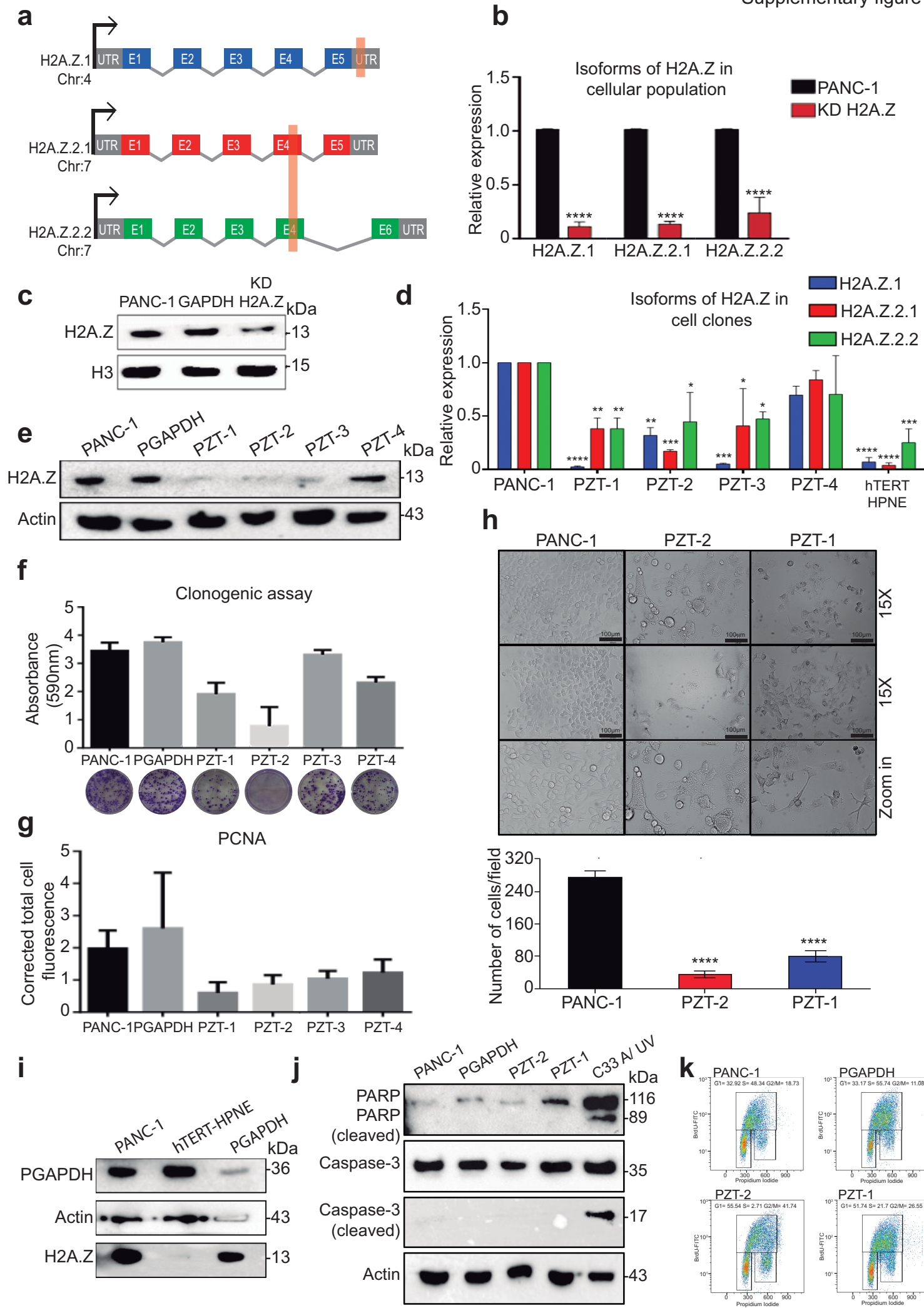

Supplement: Supplementary file 2 — Supplementary Figure 2. [file 41388_2021_1664_MOESM2_ESM.pdf]

Supplementary figure 4

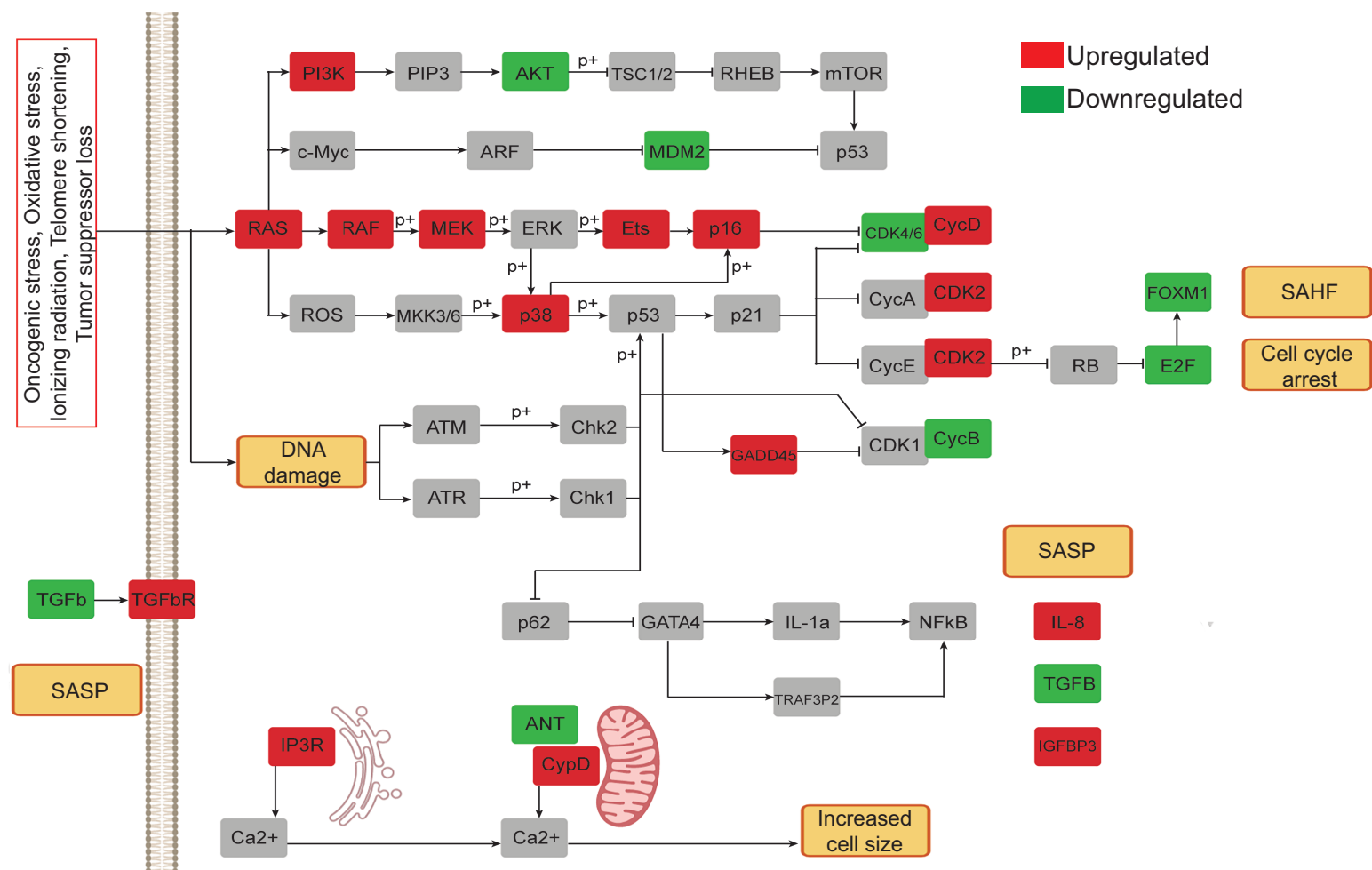

Supplement: Supplementary file 4 — Supplementary Figure 4. [file 41388_2021_1664_MOESM4_ESM.pdf]

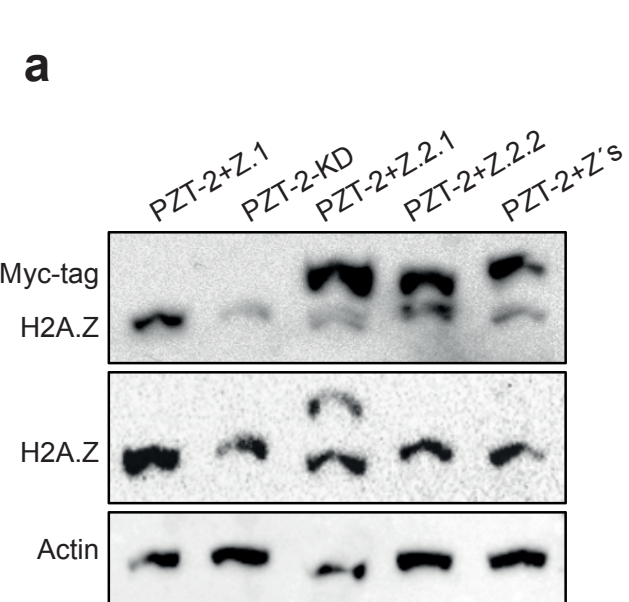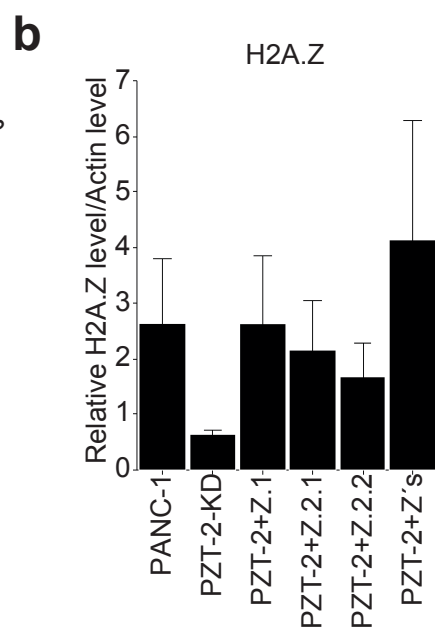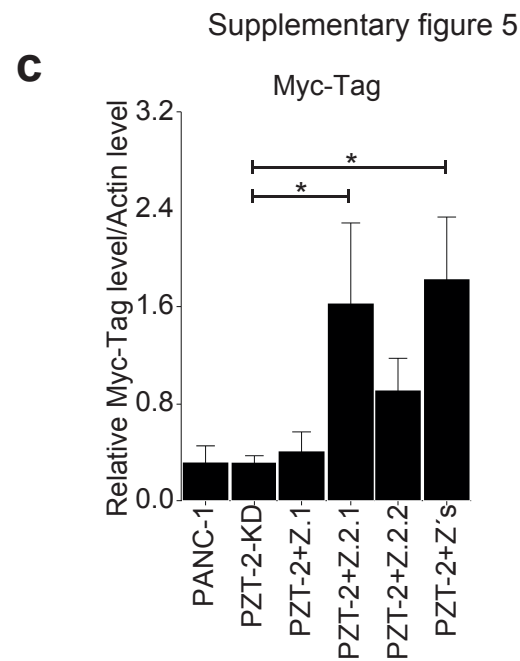

Supplementary figure 5

Supplement: Supplementary file 5 — Supplementary Figure 5. [file 41388_2021_1664_MOESM5_ESM.pdf]
