# Supplementary material for: H2A.Z overexpression suppresses senescence and chemosensitivity in pancreatic ductal adenocarcinoma
Source: Oncogene. 2021 Feb 24;40(11):2065–80. doi: 10.1038/s41388-021-01664-1 (PMC7979544; doi:10.1038/s41388-021-01664-1)

Supplementary figure 3

a

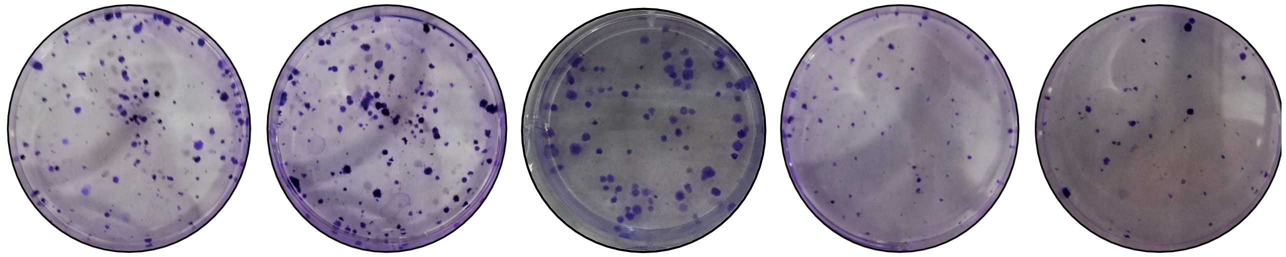

b

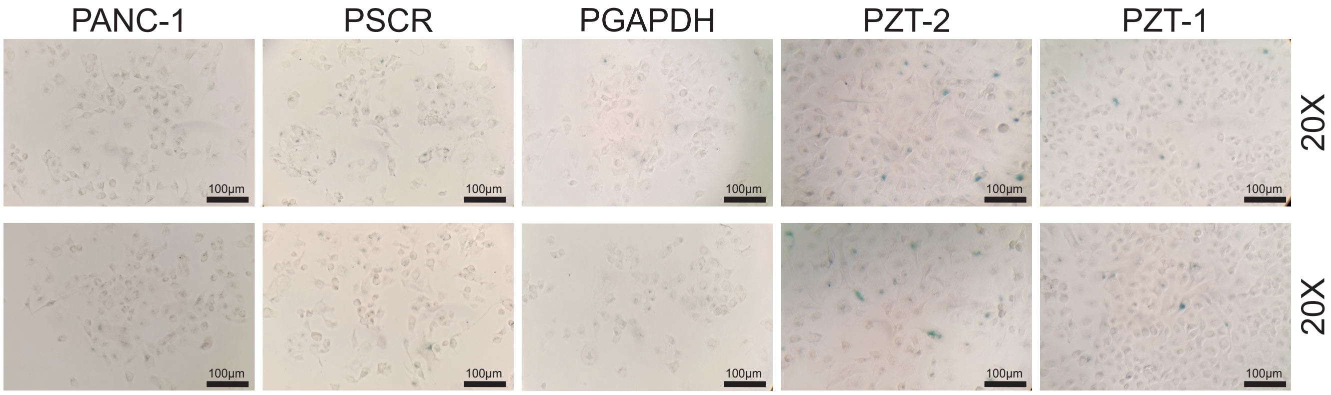

c

Term	P-value
negative regulation of cell proliferation (GO:0008285)	1.50E-04
transcription initiation from RNA polymerase II promoter (GO:0006367)	4.72E-04
positive regulation of transcription from RNA polymerase II promoter in response to stress (GO:0036003)	0.020774583
positive regulation of MAPK cascade (GO:0043410)	0.040736027
histone exchange (GO:0043486)	1.91E-07
G2/M transition of mitotic cell cycle (GO:0000086)	3.74E-07
DNA replication-independent nucleosome assembly (GO:0006336)	8.70E-07
regulation of mitotic nuclear division (GO:0007088)	2.48E-04
mitotic sister chromatid cohesion (GO:0007064)	3.08E-04
morphogenesis of an epithelial sheet (GO:0002011)	0.001582495
gene expression (GO:0010467)	0.006783067
DNA damage response, signal transduction by p53 class mediator resulting in cell cycle arrest (GO:0006977)	0.017309654
positive regulation of telomere maintenance (GO:0032206)	0.023529398
fatty acid homeostasis (GO:0055089)	0.024449542

d

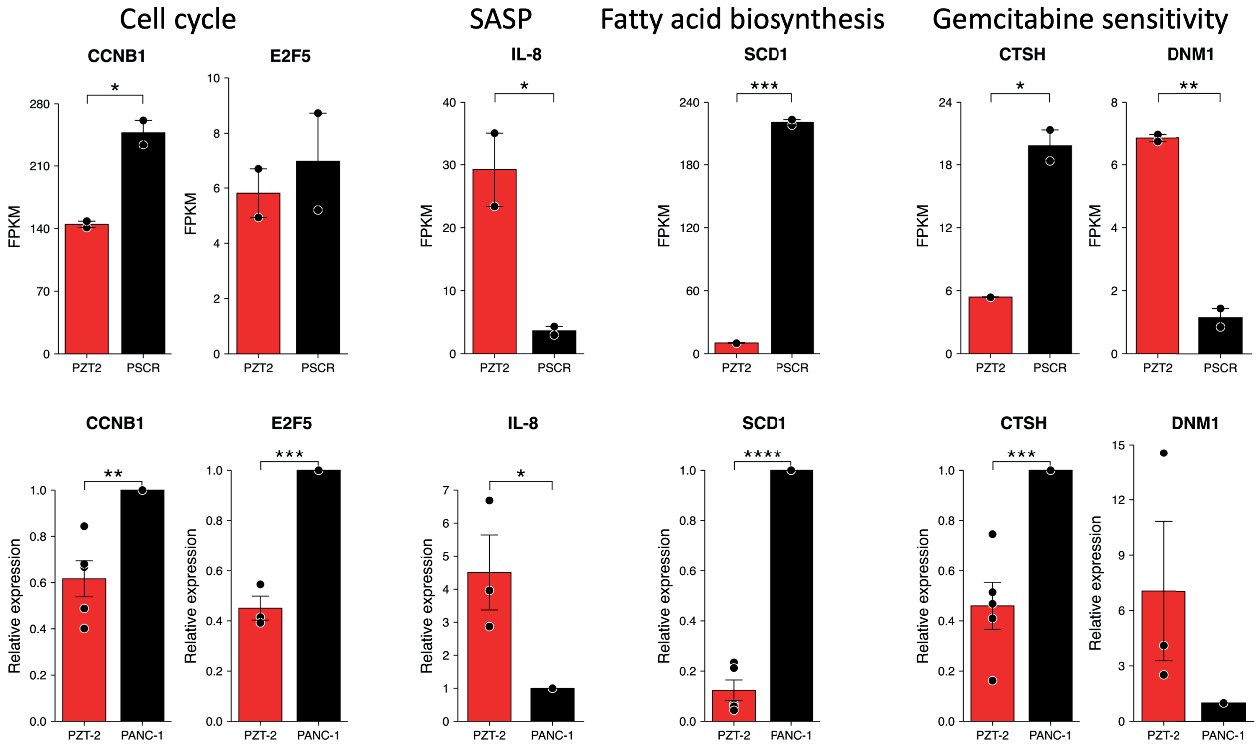

Supplement: Supplementary file 3 — Supplementary Figure 3. [file 41388_2021_1664_MOESM3_ESM.pdf]
