# Supplementary material for: H2A.Z overexpression suppresses senescence and chemosensitivity in pancreatic ductal adenocarcinoma
Source: Oncogene. 2021 Feb 24;40(11):2065–80. doi: 10.1038/s41388-021-01664-1 (PMC7979544; doi:10.1038/s41388-021-01664-1)
Supplement: Supplementary file 9 — Supplementary Figures [file 41388_2021_1664_MOESM9_ESM.docx]

**Supplementary Figures**

**Supplementary Figure 1. The isoforms of H2A.Z are highly expressed in tissues of PDAC patients.**

**a.** Normal and PDAC tissues were analyzed by IHC using anti-H2A.Z and anti-CK-7 antibodies (pink). Six representative images are shown. Scale bars = 100 (20X) or 400 μm (40X). **b.** Normal and PDAC tissues were analyzed by immunofluorescence assays (IFAs), using anti-H2A.Z (red) and anti-cytokeratin 7 antibodies (CK-7) (green). Four representative images of control and PDAC tissue are shown. Scale bar = 10 μm. **c.** Corrected total cell fluorescence of H2A.Z in IFAs. Statistical differences between two groups were evaluated by unpaired two-tailed Student's t-test. ****p- value=<0.0001

**Supplementary Figure 2. The knockdown clones decrease the expression of the three isoforms of H2A.Z at different levels.**

1. Exon/intron schematic of H2A.Z.1, H2A.Z.2.1 and H2A.Z.2.2 genes. Exons are shown as boxes, introns are shown as gray lines and UTR regions are shown as gray boxes (scheme not in real scale). The location of the shRNA sequences used to generate the lentiviral particles are showed as pale red lines (see table 3 for sequences). **b.** Expression of H2A.Z.1, H2A.Z.2.1 and H2A.Z.2.2 analyzed by RT-qPCR in the KD population normalized to untransduced PANC-1 cells. GAPDH was used as house-keeping gene. The means±SEM of three biological replicates are shown. Significance was analyzed by two-way ANOVA. ****p-value=<0.0001. **c.** Total protein extracts were analyzed by Western blot using anti-H2AZ antibody. Anti-H3 antibody was used as loading control (n=2). **d**. Depletion was confirmed by RT-qPCR in the four H2A.Z KD clones. In all of them the three isoforms of H2A.Z were decreased to different levels compared to the untransduced cell line PANC-1. The means±SEM of three biological replicates are shown. Significance was analyzed by two-way ANOVA. *p-value=<0.05; **p-value=<0.01; ***p- value=<0.001 ****p-value=<0.0001. **e.** Total extracts of the four KD clones were analyzed by western blot using an anti-H2AZ antibody. Anti-Actin antibody was used as loading control. **f** and **g**. The four KD clones show reduced proliferative capacity as determined by clonogenic assay (**f**) and PCNA staining (**g**) compared to the parental cell line PANC-1 and the control cell line PGAPDH. n=3. **h.** Cell density of PANC-1, PZT-2 and PZT-1. Two representative images are shown at 15X of the culture after 48 h of growth. Scale bar = 100 μm. The graph shows the number of cells per field for PANC-1, PZT-2 and PZT-1 and the means±SEM of 3 biological replicates. **i**. Total extracts of PANC-1 cells transduced with shRNAs against GAPDH (PGAPDH), were obtained and incubated with anti-GAPDH, anti-actin and H2A.Z antibodies antibody. Anti-actin antibody was used as loading control. **j**. Total extracts of the PANC-1 cell line, the PGAPDH cell line and the clones PZT-2 and PZT-1 were analyzed by western blot to identify expression and cleavage of caspase-3, PARP. Also, total extracts of C-33A cells in which apoptosis was induced by UV Actin was used as control. **k.** Distribution of the cell line PANC-1, PGAPDH, PZT-2 and PZT-1 in cell cycle phases G0-G1, S and G2-M as examined by staining with BrdU and IP 24 h after synchronization in G0 by serum starvation for 48 h.

**Supplementary Figure 3. Parental PANC-1 and shSCR cells behave similarly.
a.** Clonogenic capacity of the cell lines PANC-1, PSCR, PGAPDH, PZT-2 and PZT-1. **b.** SA-β-galactosidase activity in PANC-1, PSCR, PGAPDH, PZT-2 and PZT-1 show senescent cells in blue. Two representative images at 20X are shown. **c.** The gene ontology analysis in PANC-1 vs PZT-2 and PSCR vs PZT-2 cells show that the same processes were affected indicating that differently expressed genes are similar in PANC-1 vs PZT-2 and PSCR vs PZT-2. The enrichment groups have a p-value <0.05. **d.** The plots shows a normalized estimation of genes expression based on RNA-seq data for the PSCR and PTZ-2 cell lines of genes associated with cell cycle, SASP, fatty acid biosynthesis, gemcitabine sensitivity, all of them processes associate with senescence. Significance was analyzed by two-tailed Student's t test. *p =<0.05; **p =<0.01; ***p =<0.001; ****p =<0.0001.

**Supplementary Figure 4. KEGG pathway showing genes involved in the senescence pathway that are altered in the clone PZT-2**. Green rectangles depict genes with reduced transcription, red rectangles show genes with increased expression according to the transcriptome results obtained for the clone PZT-2.

**Supplementary Figure 5. Expression of the three H2A.Z isoforms analyzed by western blot in the KD clone PZT-2.** The KD clone 2 was transfected with plasmids coding for H2A.Z.1, H2A.Z.2.1 and H2A.Z.2.2 alone or together (Z’s). **a.** Total extracts of each transfected cells were obtained and analyzed by western blot using anti-H2AZ and anti Myc-Tag antibodies. Anti-actin antibody was used as loading control. **b-c.** The graph shows the relative quantification from WB for H2A.Z /Actin expression level and the expression level of Myc-Tag /Actin (respectively). The graphs depicting means SEM of 4 biological replicates. Statistical differences between the 2 groups were evaluated by unpaired two-tailed Student's t-test. * p-value ≤0.05.
