# Supplementary material for: H2A.Z overexpression suppresses senescence and chemosensitivity in pancreatic ductal adenocarcinoma
Source: Oncogene. 2021 Feb 24;40(11):2065–80. doi: 10.1038/s41388-021-01664-1 (PMC7979544; doi:10.1038/s41388-021-01664-1)
Supplement: Supplementary file 10 — Supplementary Tables [file 41388_2021_1664_MOESM10_ESM.docx]

Supplementary Tables

Supplementary Table 1. Immunohistochemistry report of H2AZ staining observed in the PDAC and control tissue samples analyzed in this study.

Supplementary Table 2. GO Biological process down-regulated

Supplementary Table 3. GO Biological process up-regulated
