# Supplementary material for: H2A.Z overexpression suppresses senescence and chemosensitivity in pancreatic ductal adenocarcinoma
Source: Oncogene. 2021 Feb 24;40(11):2065–80. doi: 10.1038/s41388-021-01664-1 (PMC7979544; doi:10.1038/s41388-021-01664-1)
Supplement: Supplementary file 11 — Supplementary Methods [file 41388_2021_1664_MOESM11_ESM.docx]

**Supplementary Methods**

**Immunohistochemistry**

Immunohistochemistry was performed as previously described ([1](#_ENREF_1)). The antibodies used were: H2A.Z (Cell signaling, 2718) at 1:100 and CK7 (Dako, M7018) at 1:100 dilution. Slides were scored by two independent pathologists (RHG and BHP) in a blinded fashion considering the percentage of H2A.Z positive neoplastic tissue and signal intensity (1, low intensity; 2, medium-low intensity; 3, medium-high intensity; and 4, high intensity). Total H2A.Z levels were determined according to the following formula: IHC score = percentage of cells positive for H2A.Z x intensity of staining ([2](#_ENREF_2)). A total IHC score <200 was considered low expression of H2A.Z and a total IHC score > 200 was considered high expression of H2A.Z.

**Protein extraction and western blot**

To obtain total protein extracts, cells were lysed in Laemmli buffer (125 mM Tris pH 6.8, 2% SDS, 20% glycerol, 10% β-mercaptoethanol and 0.01% bromophenol blue) supplemented with Complete^TM^ protease inhibitor cocktail (Roche, 1187358000) and PhosSTOP™ (Roche, 1187358000). The lysates were centrifuged at 14,000xg for 30 min at 4°C. Equal amounts (30 µg) of protein were separated by SDS-PAGE and transferred onto nitrocellulose membranes (GE Healthcare RPN303D). The membranes were blocked with fat-free milk in PBS-Tween 0.05% for 2 h at room temperature (RT) and incubated overnight with the corresponding primary antibodies (Table 4) at 4°C with constant gentle shaking. Next, membranes were washed three times with PBS-Tween 0.05% and incubated with the corresponding secondary antibodies (Table 4) at RT for 2 h. The membranes were analyzed using a ChemiDocTM MP System (BIO-RAD 1708280) using the Super Signal West Femto Maximum Sensitivity Substrate (Thermo Fisher 34095).

**RNA extraction and RT-qPCR**

Total RNA was extracted using the TRIZOL reagent ([3](#_ENREF_3)), according to manufacturer’s instructions. Total RNA was treated with RNase-free DNase-I (Thermo Fisher, EN0521). 1.5 µg of DNase-I-treated RNA were used for cDNA synthesis using 1.5 μg of oligo dT (Thermo Fisher, SO131) and 1 µL of 25 mM dNTPs, and 1µL of Super-Script II enzyme (Invitrogen, 18064014). The mixture was incubated at 42°C for 90 min and subsequently at 70°C for 5 min to inactivate the enzyme. RT-qPCR was performed in triplicates using Power SYBR^®^ Green PCR Master Mix 2X (Applied Biosystems™, 4367659). For each reaction, 100 ng of cDNA, 0.5 µL of primer mix (10 mM), and 5 µL of Power SYBR® Green PCR Master Mix 2X were mixed with water to a final volume of 10 µL. The cycle condition was: 1) Holding stage: 95ºC for 10 min, 2) cycling stage: 95ºC for 15 seg and 60ºC for 30 seg (40 cycles), and 3) melt curve stage. Gene expression was normalized to GAPDH using the ∆∆C_t_ method. Three independent biological replicates of each qPCR were performed. The oligonucleotides used are shown in Table 2.

**Gene expression data in TCGA and GTEX databases**

These databases were used to analyze the expression levels of the H2A.Z.1, H2A.Z.2.1 and H2A.Z.2.2 isoforms from PDAC patients. To analyze the expression levels of the H2A.Z.1, H2A.Z.2.1 and H2A.Z.2.2 isoforms from PDAC patients. To determine alterations in the expression of H2A.Z in PDAC patients, we used the visualization data UCSC Xena tool (<https://doi.org/10.1038/s41587-020-0546-8>) for interpreting cancer genomics data. The Transcript View showed isoform expression for both tumor tissues in The Cancer Genome Atlas (TCGA) database and normal tissues in the Genome-Type Tissue Expression (GTEX) database. All RNA-seq of both data-bases were obtained from PDAC patients and normal pancreas respectively.

**Small-hairpin interference RNA (shRNA) and transduction**

To generate stable KD of the three isoforms of H2A.Z, we generated lentiviral particles carrying shRNAs against both isoforms (Fig. 2A and Table 3) ([4](#_ENREF_4)). The shRNAs were cloned immediately downstream of the U6 promoter present in the pLL3.7-puro plasmid. Cloning of the shRNA was performed following the protocol described in <https://jacks-lab.mit.edu/protocols/psico>. Briefly, the oligonucleotides corresponding to the shRNA were aligned in a 1:1 ratio (60 pmol/µL of each oligonucleotide) in alignment buffer (100 mM K-acetate, 30 mM HEPES-KOH pH 7.4, 2 mM Mg-acetate) at 95°C for 4 min followed by an incubation at 70°C for 10 min. The pLL3.7-puro plasmid was linearized using the restriction enzymes XhoI and HpaI; and dephosphorylated using 15 U/µL of the phosphatase CIP (Amersham Pharmacia Biotech, E2250Y).

For ligation, 1µL of thermo DNA ligase (EL0011) with equimolar concentrations of the shRNA and the linearized plasmid were used and incubated at 16°C overnight. Resulting plasmids were sequenced to confirm correct insertion of the shRNA sequences. Generation of lentiviral particles was carried out by transfecting HEK293 cells using 20 µg of the pLL3.7_shRNA vector, 10 µg of the RRE expression plasmid, 10 µg of the REV expression plasmid and 10 µg of the PDM.G expression plasmid. Lentiviral particles were concentrated by ultracentrifugation at 27,000 rpm for 90 min at 4°C. For transduction, 1x10^7^ PANC-1 cells were grown to 70% confluency, lentiviral particles were added together with polybrene at a final concentration of 8 µg/mL, and the infected cells were incubated at 37°C for 2-3 days. Finally, puromycin (1µg/mL) was added to the cells. Medium was changed every 3 days for 3 weeks to select clones stably expressing the shRNA. As a negative control, PANC-1 cells were transduced with lentiviral particles carrying a shRNA against GAPDH (Table 3), an unrelated gene.

**Measurement of Caspase-3 and -7 activities**

To determine the levels of active caspase-3 and -7 we used the Caspase-Glo 3/7 assay (Caspase-GloR 3/7 assay, Promega) according to the manufacturer´s instructions. Briefly, 2,500 cells/well were seeded in a 96-well plate and incubated for 5 days. Every 24 h the activity of caspase 3 and 7 was determined by incubating 100 µL of Caspase-Glo 3/7 Reagent/well for 4 h at 37°C. The luminescence was measured in a multimode microplate reader Infinite F500 (TECAN).

**Cell viability**

To determine viable cell numbers, 5X10^3^ PANC-1 cells were seeded in 24-well plates and incubated for 5 days. Every 24 h, cells from one well were trypsinized and stained with trypan blue (0.4% trypan blue in PBS). Viable cells (trypan blue exclusion) were counted using a Neubauer chamber.

**Clonogenic assay**

Two hundred PANC-1, PGAPDH, PZT-1 and PZT-2 cells were seeded in 6-well plates and grown for 2 weeks. Cells were washed with PBS, fixed in 4% paraformaldehyde for 10 min at RT, stained with 0.5% crystal violet for 2 h at RT and washed twice with PBS. Plates were then washed thoroughly with distiller water and dried at RT. The crystal violet dye was solubilized from the cells by adding 10% acetic acid and absorbance was quantified at 590 nm using a multimode microplate reader (TECAN-infinite-**f500**-multimode-microplate-reader, 3345587).

**Incorporation of BrdU and cell cycle analysis**

Five thousand PANC-1 cells were seeded in 6-well plates and incubated for 24 h. Next, BrdU (Sigma, B9285) was added to the culture medium at a final concentration of 10 µM and incubated for 2 h at 37°C. Cells were washed with PBS and fixed with cold 70% ethanol for 30 min. Subsequently, they were incubated with a solution of 2N HCl and 0.5% Triton X-100 for 30 min at RT under rotation. Subsequently, cells were resuspended in a solution of 0.1M Na_2_B_2_O_7_ pH 8.5 for 2 h at RT and washed with PBS. Cells were incubated with blocking solution (1% BSA, 0.05% Tween 20, PBS) for 30 min. Next, 1x10^6^ cells were incubated with an anti-BrdU antibody (BD Pharmingen, 555627) at 4°C overnight. Cells were washed with 1% BSA/PBS and incubated with FITC anti-mouse secondary antibody for 2 h at 4°C in the dark. Finally, cells were washed with PBS and resuspended in PBS with 0.025 µg/mL of RNase and 2.5 µg/mL of propidium iodide and analyzed using a BD LSRII Fortessa cytometer. Data analysis was performed using FlowJo v10 software.

**SA-β-galactosidase assay**

To determine the number of senescent cells, 5,000 cells were seeded on a coverslip in a 6-well plate and incubated for 48 h. After incubation, cells were fixed with a solution of 2% formaldehyde/0.2% glutaraldehyde in PBS for 5 min at RT and washed with PBS. Next, the cells were incubated with staining solution (1 mg/ml X-Gal, 5 mM potassium ferrocyanide, 5 mM potassium ferricyanide, 150 mM NaCl, and 2 mM MgCl_2_ in H_2_O at pH 6.0) at 37°C without CO_2_ for 16 hrs. Cells were washed first with PBS then with methanol and mounted on a slide for microscopic analysis. Positive cells were counted in 5 different random fields.

**Quantification of IL-8**

To determine the concentration of IL-8 in the cell supernatant after 24 h of culture, the Human IL-8 ELISA Set kit (BD Pharmingen, 555244) was used following the supplier's instructions.

**Metabolic assay**

To determine metabolic activity, the reagent 3-(4-5-Dimethyl-2-thiazolyl)-2, 5-diphenyl-2H-tetrazolium bromide (MTT) (Sigma, M5655) was used following the manufacturer's instructions. Briefly, 1,000 cells/well were seeded per well in a 96-well plate and incubated for 5 days. Every 24 h the metabolic activity was determined by incubating the cells with 10 µL of MTT Reagent/well for 4 h at 37°C followed by addition of 100 µL of Detergent Reagent (4mM HCl, 0.1% NP-40, isopropanol) and overnight incubation at 37°C in the dark. The measured absorbance at 570 nm corresponds to metabolic activity of each sample.

**RNA-seq**

The integrity of total RNA samples for the preparation of the RNA-seq libraries was determined by Automated Capillary Electrophoresis using the Bioanalyzer 2100 system (Agilent Technologies, Inc., Santa Clara, CA) and the Agilent RNA 6000 Nano chip. All values of RNA integrity obtained for the samples were >8. mRNAs were enriched using magnetic beads (Illumina). Construction of the RNA-seq libraries was performed using the Illumina TruSeq Stranded mRNA sample prep kit (Illumina, Inc., San Diego, CA) according to the manufacturer’s instructions. Subsequently, the RNA was fragmented in a range of 200-600 bp. Both ends of the double-stranded cDNA were repaired using T4 DNA polymerase and T4 DNA ligase, respectively, to subsequently bind the specific Illumina adapters. Finally, each library was amplified by PCR using specific oligonucleotides for the P5 and P7 adapters of Illumina to increase the number of molecules for massive sequencing. Each library was analyzed by Automated Capillary Electrophoresis (Bioanalyzer 2100, Agilent Technologies, Inc., Santa Clara, CA) using the DNA High Sensitivity chip. The libraries were quantified using the Qubit® fluorometer with the Qubit ds DNA HS Assay kit (Invitrogen). The libraries were clonally amplified using Flowcell and sequenced using the NextSeq 500 platform (Illumina, Inc., SanDiego, CA) and the NextSeq 500/550 Mid Output kit v2 (150 cycles) with a Paired-End 2X76 run configuration according to the manufacturer’s protocols.

**Differential transcript expression analysis clones**

A total of 35,205,405 and 34,046,499 paired-end RNA-seq reads for PANC1 wild type cells, PSCR cells and PZT-2 knockdown cells, respectively, were adapter- and quality-trimmed using Trim Galore version 0.4.1. Transcript-level abundance was estimated using Kallisto (<https://pachterlab.github.io/kallisto/>) version 0.43.0 ([5](#_ENREF_5)). Quantification algorithms were run on paired-end RNA-seq files from the biological samples using an index created from a FASTA file containing the RefSeq annotated transcripts with bootstraps parameter set at 100 (-b 100). Kallisto output abundance files were analyzed to calculate differential expression, using Sleuth (<https://pachterlab.github.io/kallisto/>) version 0.29.0 ([6](#_ENREF_6)). Wald tests were performed to establish expression patterns by comparing PZT-2 versus PANC-1 wild type samples.

**Gene Ontology and Gene Set Enrichment Analysis**

To determine which biological processes are affected by depletion of the three H2A.Z isoforms, we used The Database for Annotation, Visualization and Integrated Discovery (DAVID) v6.8. (https://david.ncifcrf.gov/) and Kyoto Encyclopedia of Gene and Genomes (KEGG; https://www.genome.jp/kegg/). To determine alterations in genes involved in cellular senescence, we used Gene Set Enrichment Analysis (GSEA v3.0) GO_CELLULAR_SENESCENCE, (Systematic name: M11558). To determine the transcription factors involved in the regulation of the altered genes in PZT-2 cells, we used the ChEA analysis ([7](#_ENREF_7)). A *p*-value <0.05 was considered as a cut-off point for the enrichment analysis.

**Cytotoxicity assays**

One thousand PANC-1 cells/well were seeded in 96-well plates and incubated for 24 h. Next, logarithmic concentrations of gemcitabine (Sigma 6423) (1,000, 100, 10, 1, 0.1, 0.01, 0.001 and 0 µM) were added and incubated for 72 h at 37°C. Finally, 10 µL of MTT Reagent/well was added for 4 h at 37°C. Subsequently, 100 µL of Detergent Reagent (4mM HCl, 0.1% NP-40, isopropanol) was added and incubated at 37°C overnight in the dark. Absorbance was determined at 570 nm and curves of sensitivity to gemcitabine and the IC_50_ values were calculated using GraphPad Prism v6.01 software (GraphPad Software, San Diego, CA).

**Xenograft mouse model**

The Institutional Animal Care and Use Committee (IACUC) of CINVESTAV approved all animal experiments (protocol number: 0258-17). Three male 5-6 weeks old nu/nu mice were used in the experiments. Five million PANC-1, PZT-2 or PZT-1 cells resuspended in PBS were injected subcutaneously into the right flanks.

Mice were examined 3 times per week for tumor growth. Once the tumor reached a size of 3,000 mm^3^, mice were sacrificed by cervical dislocation and the tumors were extracted, fixed in formalin and embedded in paraffin for histological analysis. The tumor size was calculated using the following formula: Tumor volume = (long diameter x short diameter^2^)/2.

**Overexpression assay**

To rescue the oncogenic phenotype in the KD clone PZT-1 and PZT-2, we over-expressed the H2A.Z isoforms using the plasmids pcDNA3-H2A.Z.1, pCMV6-H2A.Z.2.1-Myc/DDK (TrueORFGold, RC200564) and pCMV6-H2A.Z.2.1-Myc/DDK (TrueORFGold, RC217665). As a negative control, the plasmid pcDNA3 was used. Briefly, 5x10^4^ cells of clones PZT-1, PZT-2 or PANC-1 cells were seeded in 6-well plates and incubated at 37°C for 24 h. When the cells reached 70% confluence, they were transfected with 1 µg of plasmid and 2 µl/ml of TurboFectTM (Thermo, R0533), and incubated at 37°C. Transfected cells were used for RT-qPCR, resistance to gemcitabine, and SA-β-galactosidase assays 48 h post-transfection, as described above.

**References**

1. Cruz-Nova P, Schnoor M, Correa-Basurto J, Bello M, Briseno-Diaz P, Rojo-Dominguez A, et al. The small organic molecule C19 binds and strengthens the KRAS4b-PDEdelta complex and inhibits growth of colorectal cancer cells in vitro and in vivo. BMC Cancer. 2018;18(1):1056.

2. Cederbye CN, Palshof JA, Hansen TP, Duun-Henriksen AK, Linnemann D, Stenvang J, et al. Antibody validation and scoring guidelines for ABCG2 immunohistochemical staining in formalin-fixed paraffin-embedded colon cancer tissue. Sci Rep. 2016;6:26997.

3. Chomczynski P, Sacchi N. Single-step method of RNA isolation by acid guanidinium thiocyanate-phenol-chloroform extraction. Anal Biochem. 1987;162(1):156-9.

4. Vardabasso C, Hake SB, Bernstein E. Histone variant H2A.Z.2: A novel driver of melanoma progression. Mol Cell Oncol. 2016;3(2):e1073417.

5. Bray NL, Pimentel H, Melsted P, Pachter L. Near-optimal probabilistic RNA-seq quantification. Nat Biotechnol. 2016;34(5):525-7.

6. Pimentel H, Bray NL, Puente S, Melsted P, Pachter L. Differential analysis of RNA-seq incorporating quantification uncertainty. Nat Methods. 2017;14(7):687-90.

7. Lachmann A, Xu H, Krishnan J, Berger SI, Mazloom AR, Ma'ayan A. ChEA: transcription factor regulation inferred from integrating genome-wide ChIP-X experiments. Bioinformatics. 2010;26(19):2438-44.
